# Differential Responses of Brain, Gonad and Muscle Steroid Levels to Changes in Social Status and Sex in a Sequential and Bidirectional Hermaphroditic Fish

**DOI:** 10.1371/journal.pone.0051158

**Published:** 2012-12-10

**Authors:** Varenka Lorenzi, Ryan L. Earley, Matthew S. Grober

**Affiliations:** Department of Biology and Center for Behavioral Neuroscience, Georgia State University, Atlanta, Georgia, United States of America; University of Minnesota, United States of America

## Abstract

Sex steroids can both modulate and be modulated by behavior, and their actions are mediated by complex interactions among multiple hormone sources and targets. While gonadal steroids delivered via circulation can affect behavior, changes in local brain steroid synthesis also can modulate behavior. The relative steroid load across different tissues and the association of these levels with rates of behavior have not been well studied. The bluebanded goby (*Lythrypnus dalli*) is a sex changing fish in which social status determines sexual phenotype. We examined changes in steroid levels in brain, gonad and body muscle at either 24 hours or 6 days after social induction of protogynous sex change, and from individuals in stable social groups not undergoing sex change. For each tissue, we measured levels of estradiol (E_2_), testosterone (T) and 11-ketotestosterone (KT). Females had more T than males in the gonads, and more E_2_ in all tissues but there was no sex difference in KT. For both sexes, E_2_ was higher in the gonad than in other tissues while androgens were higher in the brain. During sex change, brain T levels dropped while brain KT increased, and brain E_2_ levels did not change. We found a positive relationship between androgens and aggression in the most dominant females but only when the male was removed from the social group. The results demonstrate that steroid levels are responsive to changes in the social environment, and that their concentrations vary in different tissues. Also, we suggest that rapid changes in brain androgen levels might be important in inducing behavioral and/or morphological changes associated with protogynous sex change.

## Introduction

Sex steroids have an important role in modulating social behavior [1], and social interactions can, in turn, influence steroid levels [2], [3], [4], [5]. For instance, territorial challenges simulated by acoustic playbacks increase 11-ketotestosterone (KT), the most potent fish androgen, in Gulf toadfish, *Opsanus beta* [6] and non-invasive administration of KT, in turn, modulates male calling behavior [7]. Although the vast majority of studies on steroids and aggression focus on males, female responses do not always align with those of males. Female song sparrows *Melospiza melodia* show decreased testosterone (T) in response to territorial intrusion [8] while female California mice *Peromyscus californicus* show no change in T [9]. In the cooperative breeding cichlid *Neolamprologus pulcher,* female and male residents and intruders have higher T and KT than controls that did not receive a territorial challenge, but only male aggression correlates with T [10]. Estrogen levels in these females were not affected by social challenge [9], [10], [8]. All of these studies examined changes in circulating steroids, but plasma levels can be different from local tissue levels [11]. While the gonads are an important source of steroids and probably a major contributor to circulating levels, there is evidence that a variety of tissues, including the brain, also are capable of steroidogenesis. Homogenates of brain tissues from goldfish *Carassius auratus*, toadfish, and Atlantic salmon *Salmo salar* parr produced large amounts of estrogen, and ovaries and testes synthesized much less estrogen than brain tissue [12], [13]. In fish, male blood KT levels are typically high, but there is evidence that this could be due to extragonadal production: after castration, implanting male sticklebacks *Gasterosteus aculeatus* with the KT precursor 11-ketoandrostenedione (OA) produced high plasma KT levels [14]. In male rainbow trout *Oncorhynchus mykiss*, highest OA to KT conversion activity was found in liver, testis and kidney, followed quite closely by intestine, brain and spleen, while OA activity in muscle was negligible [15]. Because muscle is not considered an important site of steroidogenesis, its steroid levels should be reflecting circulating levels, and so we included it in our analysis as a type of negative control.

We measured steroids in the brain because local synthesis of steroids could affect behavior through fast, non-genomic mechanisms that can act in a time frame more consistent with rapid behavioral change [16], [17], [18], [19], [20], [21]. *In vivo* microdialysis of male zebra finch *Taeniopygia guttata* brain showed local changes in steroid levels in response to social stimuli; playback of male songs caused an increase in estradiol (E_2_) and a decrease in T while presenting females to males caused an increase in E_2_ but not T [22]. These changes in brain steroid concentrations were not reflected in plasma steroid levels [22]. Some studies have found a correlation between plasma and tissue steroid levels (see below), but few have examined hormone concentrations across different tissues within an individual. For example, there is a strong correlation between blood and mucus KT, but not between blood and muscle KT in koi *Cyprinus carpio* [23]. In the ratfish *Hydrolagus colliei*, there is a significant positive correlation between plasma and muscle T and KT, but not E_2_ [24]. A study on the protogynous *Thalassoma duperrey* showed some discrepancies between gonad and plasma steroids [25]. *In vitro* gonad culture showed that testes of territorial dominant males produced much more KT than females and newly sex changed males, and gonads of sex changed males produced more KT than female gonads. When comparing plasma KT, however, neither types of male differed from females. Testosterone production by the gonad was higher in all males relative to females, but there was no significant sex difference in plasma T levels [25].

Given the mounting evidence that circulating steroid levels are not always concordant with tissue specific levels, we tested whether steroid levels in brain, gonad and muscle show sex or status differences in steroid concentrations and, if so, whether they are differentially responsive to changes in the social environment in the bluebanded goby *Lythrypnus dalli*, a bidirectional sequential hermaphrodite. Sex change in this species is controlled by social status [26], [27], and their small size allows for the expression of natural behavior and sex reversal in a controlled laboratory setting. In *L. dalli*, females can be as aggressive as males and form linear dominance hierarchies [27], so it is also an ideal species for examining differences in steroids among females within different status classes. Natural changes in or manipulation of sex steroids are associated with sex change (reviewed by Frisch [28]). In protandrous species, E_2_ levels increase with transition from male to female, and administering E_2_ to males induces sex change. In protogynous species, KT levels increase during sex change, and KT administration can induce gonadal sex change in females. Testosterone in many species does not vary during sex change. The vast majority of these studies on sex change are based on ‘circulating’ levels of hormone measured from whole body homogenates [29], [30], plasma [31], [32], [33], [34], [35], [36], [37] or fish holding water (Earley et al. unpublished data).

**Figure 1:**
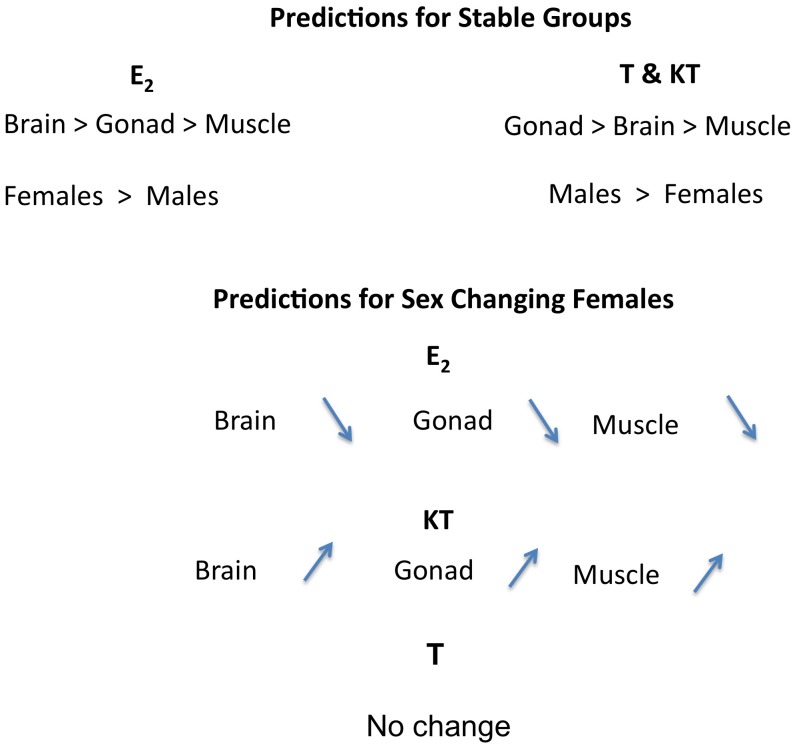
Experimental predictions. If steroid concentrations in tissue were simply determined by circulating levels, then we would expect all of them to change in similar ways during sex change.

In the present experiment, we tested the hypothesis that tissue hormone concentrations are different between males and females, and that E_2_ drops and KT increases during sex change (Fig.1). Our null prediction is that the hormonal profiles of all tissues would show the same directional change in response to changes in the social environment. Deviations from this null prediction would suggest that different mechanisms control hormone concentrations in different tissues.

## Materials and Methods

### Ethics Statement

The research conducted herein was approved by the Georgia State University IACUC protocol No. A06030.

### Experimental Design

*Lythrypnus dalli* is a small benthic marine goby (20–50 mm adult standard length) in which large males typically dominate a nesting territory containing multiple females [38]. Under laboratory conditions, these fish form linear dominance hierarchies when kept in social groups similar to those found in nature [27]. The fish used in this study were collected off the coast of Catalina Island, CA, in June 2007 by SCUBA diving (permit #803036-03 to VL), and were initially housed in communal holding tanks at the Wrigley Institute of Environmental Sciences on Catalina Island. From these animals, thirty experimental social groups were established in indoor seawater tables with continuous seawater supply and exposure to the natural summer light cycle. Each social group consisted of a male (the largest animal in the tank), a large (alpha) female, a medium-sized (beta) female that was at least 3 mm smaller than alpha, and a small (gamma) female; these size differences ensure stable hierarchy positions. Behavioral observations consisted of 10 min sessions, and we recorded the following behaviors: approaches, displacements received, displacements given, attacks, and threat displays. Approach behavior is defined as a fish moving within one body length of another fish, and can be considered a form of challenge to the other fish; the fish being approached can react or not, and this often depends on its social status. If the approach ends with the subordinate fish moving away, then it is scored as a displacement (see below) and is a good measure of relative dominance. The attack is a very quick approach aimed directly towards the other fish and causing the fish that was attacked to rapidly move away. Attacks happen very fast and if the subordinate fish is too slow to escape, the attack can end with physical contact. Conversely, displacements are much less aggressive in nature and describe situations where the dominant fish just swims in the proximity of the subordinate fish, which moves away. Submission is measured as the number of times a fish moves away from another member of the social group in response to approaches, attacks, or displacements. Finally, threat displays occur when two fish approach one another raising the dorsal fin and opening the opercula while swimming sideways or in circle.

In ten groups, fish were kept in the tank for 7 days without any social manipulation, and these are referred to as “stable” groups. Behavior was observed on day 6 in the morning and in the afternoon, and on day 7 in the morning. Tissue samples were collected from all group members (male and all females) on day 7. In the other twenty social groups, the male was removed from each tank after 6 days to induce sex change in the alpha female. Behavior was observed on day 5 in the morning and in the afternoon, on day 6 in the morning plus one hour after male removal in the afternoon, and on day 7 (the day after male removal) in the morning. In ten of these groups, tissues were collected from the alpha and beta females 24 hours after male removal, and these will be referred to as “sex change 24 h” groups. In the remaining ten groups, tissues were collected from alpha and beta females 6 days after male removal. In these groups, additional behavioral observations were performed 5 days after male removal in the morning and in the afternoon, and 6 days after male removal in the morning. These will be referred to as “sex change 6d” groups. For each fish, we recorded standard length, and captured a digital image of the external genitalia (genital papilla) at the start and at the end of the experiment. The shape of the genital papilla is a reliable indicator of the sex of the fish and is expressed as length/width ratio. A ratio of 1.2 or less is typical of a round female papilla, while a ratio >1.6 is typical of a thin pointed male papilla; transitional fish have intermediate values [27]. We used AxioVision (Zeiss Inc.) imaging software to take papilla measurements from digital images. Fish were then killed via exposure to a lethal dose of tricaine methanesulfonate (MS222). Brain, gonads and caudal muscle were collected, and a digital image of the gonads was taken to confirm sex and reproductive status. Each tissue was put in a microcentrifuge vial and immediately frozen in liquid nitrogen. All the fish were killed between 1400 and 1600 h. Previous work showed that *L. dalli* water-borne androgen levels peak in the morning and E_2_ levels peak in the evening [39], so we assayed tissues when hormones do not fluctuate dramatically to avoid differences due to diurnal rhythms. The tissue samples were shipped frozen to Georgia State University and stored at −80°C until processing for steroid content.

### Steroid Analysis

Steroid hormones were extracted from tissue following a solid-phase extraction protocol (modified from [40]. Briefly, each frozen tissue sample was weighed, transferred to borosilicate vials, and homogenized in 350 µl of buffer in an ice-cold water bath (0.1 M phosphate buffer for brain and gonad samples [pH 7.5], 0.1 M borate buffer [pH 7.5] for muscle samples). After homogenization, 1.5 ml methanol was added to each sample, and they were quickly vortexed and returned to the ice bath. When all samples were homogenized, they were vortexed for 60 min and then stored at 4°C overnight. We repeated the same protocol for 2 control blank samples without any tissues: one containing 0.1 M phosphate buffer and one with 0.1 M borate buffer. The next day, samples were vortexed again for 20 min at room temperature, and centrifuged at 1027*×g* for 10 min at 4°C. The supernatant was decanted, and 16 ml of water was added to dilute the methanol. The homogenized samples were connected through high purity tubing (Saint-Gobain Tygon formulation 2275) to Sep-Pak C18 columns (Waters) mounted onto a vacuum manifold. Before starting, the columns were primed with 2×2 ml washes of methanol followed by 2×2 ml washes of distilled water. After drawing the samples through the columns, the columns were rinsed with 2×2 ml of distilled water to purge salts, and then total hormones were eluted with 2×2 ml of methanol. The eluted methanol was collected in borosilicate vials, dried under a stream of nitrogen, and the resulting hormone residue was resuspended in 350.5 µl of a solution made of 95% EIA buffer and 5% ethanol. After vortexing for 15 min, samples were stored at 4°C overnight. Samples were vortexed for 45 min prior to assay. EIA kits (Cayman Chemicals, Inc.) for E_2_, KT and T were used to quantify hormone in the samples; kit protocols were strictly followed. Assay plates were read on a spectrophotometer (Opsys MR, Dynex Technologies) at a wavelength of 405 nm after 90 min of incubation. See supporting information text for details on the validation of the hormone assays.

### Statistical Analysis

For stable groups, we compared hormone levels among males and alpha, beta and gamma females. We could not perform a multivariate analysis because levels of E_2_, T, and KT in each fish are not independent, so we performed three separate ANOVAs, one for each hormone. We tested for normality and homogeneity of variance. To achieve normality, T and E_2_ levels were log transformed and KT was square root transformed. When the main effects in our statistical models were significant, we performed Tukey’s HSD post hoc tests to evaluate differences among the levels. For interaction terms, the number of possible comparisons were numerous, but only a subset of them were relevant to our hypotheses. We thus generated *a priori* linear contrasts to evaluate differences among the levels of our interaction terms. For sex changing groups, we performed two different analyses. In the first, we performed ANOVAs comparing hormone levels of alpha (dominant) females initiating sex change after male removal at 24 hours and 6 days after male removal to alpha females of stable groups with the male present, and beta-ranking females ascending to dominant female position in sex-changing groups to beta females in stable groups with the male present. This analysis tests whether steroid profiles in sex-changing alphas deviated significantly from stable alphas and whether betas whose status was elevated to alpha differ from individuals who remain beta. In the second analysis, we performed ANOVAs to compare alphas in the sex-changing groups to males, and betas in the sex changing groups to alphas in stable groups. This analysis tests whether steroid profiles in sex-changing alphas approached the profile of stable males, and whether betas whose status was elevated exhibit steroid profiles comparable to those of established alphas. Paired t-tests were used to compare values of papilla ratio for each animal at the start versus the end of the experiment. We performed correlations between behavior and hormone levels and between hormones in different tissues. The ANOVAs and linear contrasts were performed with SAS version 8.1 (SAS Institute Inc.), while the correlation analyses were performed with JMP (SAS Institute Inc.). All data are reported as mean ± SEM.

## Results

### Genital Papilla

None of the fish in stable groups changed sex, based upon the shape of their external genitalia. Initial and final genital papilla length/width ratios were not significantly different for males (paired t test: t_9_ = 1.33, p = 0.215; initial = 3.28±0.20; final = 3.50±0.15), alphas (paired t test: t_9_ = 1.29, p = 0.229; initial = 1.09±0.04; final = 1.12±0.04), betas (paired t test: t_9_ = 0.64, p = 0.540; initial = 1.09±0.04; final = 1.12±0.04) or gammas (paired t test: t_9_ = 0.90, p = 0.394; initial = 1.13±0.07; final = 1.16±0.03) from stable groups.

Alpha females in sex change treatments did not show any significant changes in the papilla ratio 24 h after male removal (paired t test: t_8_ = 1.39, p = 0.202; initial = 1.12±0.02; final = 1.16±0.03), but the papilla shifted towards the male typical shape 6d after male removal (paired t test: t_9_ = 2.51, p = 0.033; initial = 1.17±0.04; final = 1.38±0.07). Beta females in sex change treatments did not show any significant changes in the papilla ratio 24 h (paired t test: t_8_ = 0.67, p = 0.523; initial = 1.08±0.02; final = 1.11±0.02) or 6d after male removal (paired t test: t_9_ = 0.54, p = 0.601; initial = 1.16±0.03; final = 1.18±0.02).

### Hormones in Stable Groups

Because of the large number of statistical comparisons conducted in the following sections, we generated a full list of significant linear contrasts for stable groups in Table S1 of the supporting information. All results in this section refer to Table S1 unless otherwise noted.

#### Estradiol

**Figure 2:**
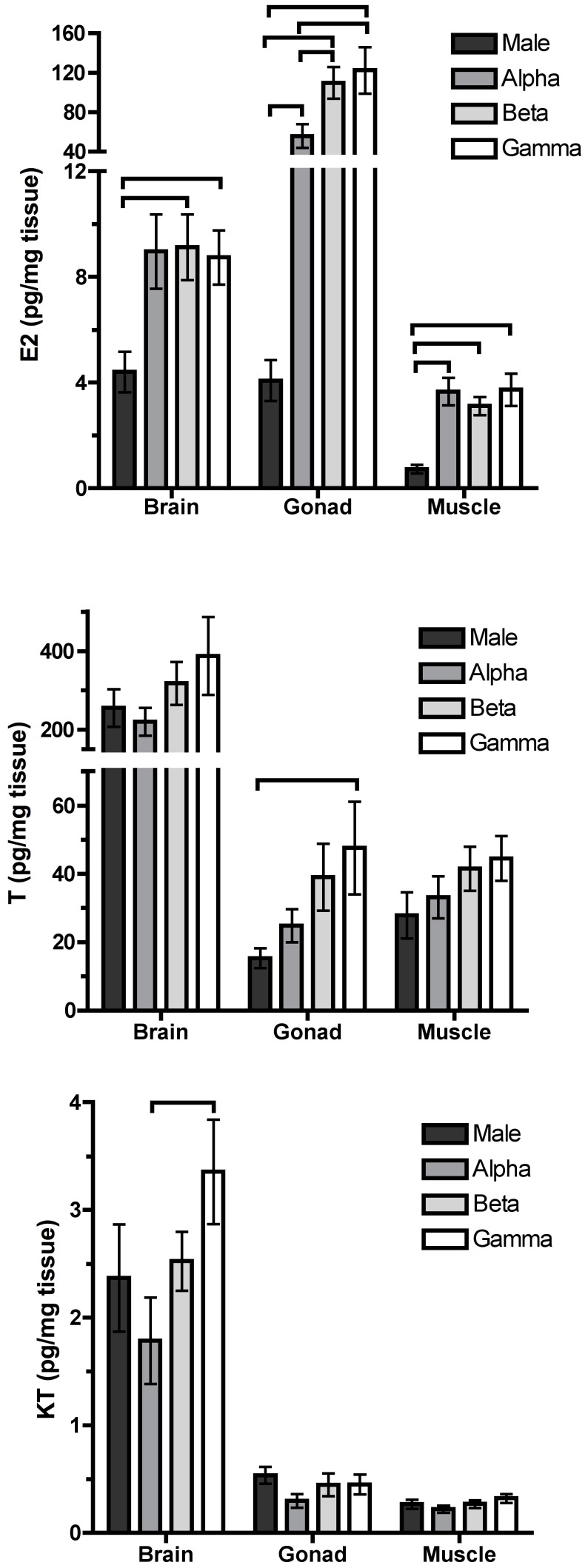
Steroid concentrations in stable groups. Concentrations of estradiol (E_2_), testosterone (T) and 11-ketotestosterone (KT) in stable groups. Lines between bars represent significant differences.

There were significant status, tissue, and status×tissue effects on E_2_ concentrations (Table 1). Overall, females had significantly higher E_2_ than males. Linear contrasts revealed that for all tissues, alpha, beta, and gamma females had significantly higher E_2_ than males. Beta and gamma females had significantly higher gonad E_2_ than alphas. In females, gonads had significantly higher E_2_ than the other tissues, and brain had significantly more than muscle (Fig. 2). In males, gonad and brain E_2_ did not differ but both had significantly higher E_2_ than muscle (Fig. 2).

**Table 1:** Summary of ANOVAs comparing sex steroids across status classes and tissue types.

	Estradiol		Testosterone		11-Ketotestosterone	
	F_df_	P	F_df_	P	F_df_	P
**Stable Groups**						
Status	F_3,104_ = 71.8	P<0.0001	F_3,105_ = 7.2	P = 0.0002	F_3,106_ = 5.77	p = 0.0011
Tissue	F_2,104_ = 224.5	P<0.0001	F_2,105_ = 148.2	P<0.0001	F_2,106_ = 168.8	P<0.0001
Status×Tissue	F_6,104_ = 12.1	P<0.0001	ns	ns	ns	ns
**Sex Changing Groups; Alpha vs Beta Females**						
Time	F_2,143_ = 3.2	P = 0.045	F_2,149_ = 157.0	P<0.0001	ns	ns
Status	F_1,143_ = 12.4	P = 0.0006	F_1,149_ = 13.4	P = 0.0003	ns	ns
Tissue	F_2,143_ = 515.9	P<0.0001	F_2,149_ = 192.1	P<0.0001	F_2,152_ = 342.5	P<0.0001
Time×Status	F_2,143_ = 3.5	P = 0.033	ns	ns	F_2,152_ = 6.7	P = 0.0016
Time×Tissue	ns	ns	F_4,149_ = 24.7	P<0.0001	F_4,152_ = 8.7	P<0.0001
Status×Tissue	ns	ns	ns	ns	ns	ns
Time×Status×Tissue	F_4,143_ = 3.5	P = 0.0097	ns	ns	ns	ns
**Sex Changing Groups; Alpha Females vs Males**						
Time	F_2,145_ = 52.2	P<0.0001	F_2,149_ = 111.6	P<0.0001	ns	ns
Status	F_1,145_ = 90.0	P<0.0001	F_1,149_ = 4.1	P = 0.044	F_1,152_ = 12.5	P = 0.0005
Tissue	F_2,145_ = 357.3	P<0.0001	F_2,149_ = 194.9	P<0.0001	F_2,152_ = 311.2	P<0.0001
Time×Status	F_2,145_ = 30.4	P<0.0001	ns	ns	ns	ns
Time×Tissue	F_4,145_ = 13.2	P<0.0001	F_4,149_ = 29.0	P<0.0001	F_4,152_ = 8.9	P<0.0001
Status×Tissue	F_2,145_ = 7.7	P = 0.0007	F_2,149_ = 3.4	P = 0.038	ns	ns
Time×Status×Tissue	F_4,145_ = 5.9	P = 0.0002	ns	ns	ns	ns

#### Androgens

For both T and KT, there were significant main effects of status and tissue (Table 1). Beta and gamma females had more T than males, and gammas had more T and KT than alpha females (Tukey’s HSD, p<0.05). Brain had significantly higher T and KT than muscle and gonad (Tukey’s HSD, p<0.05; Fig. 2). Ovaries of females pooled across status classes had significantly higher T than testes (Table S1; female gonad vs. male gonad). This effect was driven by gammas and betas whose gonads had significantly more T than male gonads (Table S1 and Fig. 2).

### Hormones in Sex Changing Groups: Comparing Alpha and Beta Females

We compared hormone levels of alpha females in stable groups with those of sex-changing alphas and hormone levels of beta females in stable groups with those of betas rising to the alpha female position at 24 h and 6d after male removal. The full list of significant linear contrasts and their statistics are reported in Table S2, and a summary of the three-way interaction for E_2_ in Table S3 of the supporting information. All results in this section refer to Table S2 unless otherwise noted.

#### Estradiol

**Figure 3:**
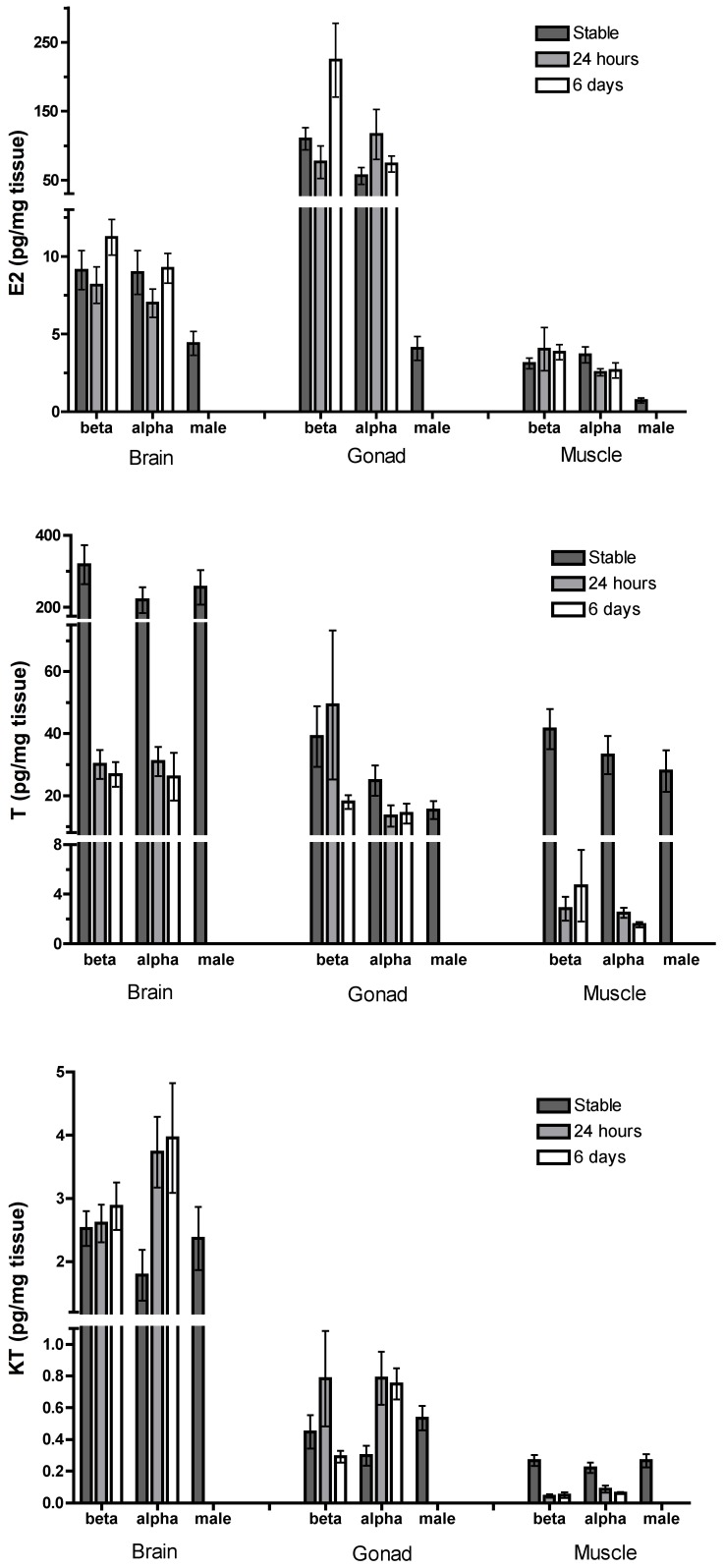
Steroid concentrations in sex changing groups. Concentrations of estradiol (E_2_), testosterone (T) and 11-ketotestosterone (KT) in stable and sex changing groups. The legends “24 hours” and “6 days” refer to time after male removal.

Estradiol varied significantly with time, status, and tissue in the sex changing groups (Table 1). The significant three-way interaction (time×status×tissue) was driven largely by differences in E_2_ among tissue types. Linear contrasts revealed significantly higher E_2_ in the gonad than in brain and muscle (Fig. 3), and in brain than muscle for alphas (stable and sex-changing) and betas (stable and rising to alpha position) at all time points (Table S3). Betas rising to the alpha position had significantly higher E_2_ (pooled tissues) at 6d than at 24 h and compared to beta females of stable groups; this significant difference was likely driven by gonad E_2_, which was the only tissue to vary with time (Fig. 3 and Table S3). Six days after male removal, betas rising to the alpha position had significantly higher E_2_ than sex-changing females, which also appeared to be driven by differences in gonad E_2_ (Fig. 3 and Table S3).

#### Androgens

Testosterone was higher in betas (stable and rising to alpha position) than alphas (stable and sex-changing), an effect driven mainly by gonad T (Fig. 3). Sex-changing females had higher KT at 24 h and 6d after male removal than in alpha females of stable groups. For both alphas (stable and sex-changing) and betas (stable and rising to alpha position), KT was significantly higher in brain than in gonad and muscle while brain T was significantly higher than gonad in stable and 6d groups and higher than muscle at all time points (Fig. 3). Alpha and beta females of stable groups had higher brain and muscle T than alphas and betas from sex changing groups, respectively, at the 24 h and 6d time points (Fig. 3). Brain KT was significantly higher at 24 h and 6d after male removal than in stable groups, but only at 24 h in the gonad (Fig. 3). In muscle, KT was higher in stable groups than after male removal.

### Hormones in Sex Changing Groups: Comparing with Males

We compared hormone levels of sex-changing females (alphas) with those of males, and hormones of betas rising to alpha position at 24 h and 6d after male removal with those of dominant alpha females in stable groups (Table 1). Males had significantly lower E_2_ than sex-changing females (Fig. 3). Beta females rising to alpha position had significantly higher gonad E_2_, and lower brain and muscle T than dominant females in stable groups (Fig. 3). Sex-changing females had significantly higher brain KT than males, but lower muscle KT and lower brain and muscle T (Fig. 3). Due to space constraints, we present the details of this result section in the text of the supporting information, the full list of significant linear contrasts in Table S4, and a summary of the three-way interaction for E_2_ in Table S5 of the supporting information.

### Hormone-by-Hormone Correlations

**Figure 4:**
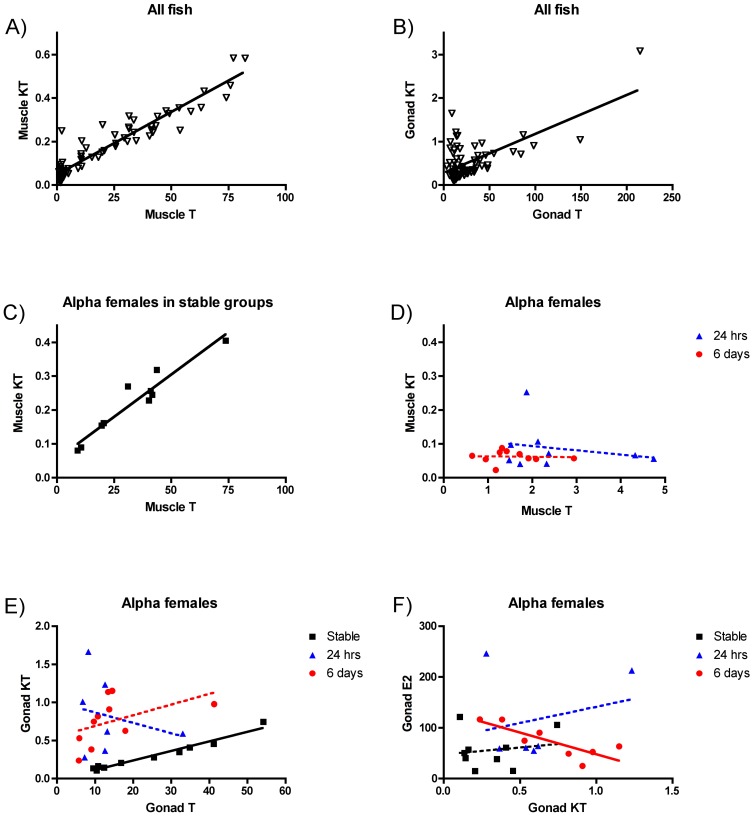
Correlations between different steroids. Correlations between concentration of KT and T in the muscle (A) and in the gonad (B) across all fish, and in the muscle of alpha females in stable groups (C) and in sex changing groups (D). Correlations between concentration of KT and T (E) and between E_2_ and KT in the gonad of alpha females. A solid line means that the associated p value is significant (p<0.05) while dotted lines are associated with non-significant correlations.

We examined the relationship between tissue steroid concentrations and due to the large number of comparisons we report details in Table 2. All results in this section refer to Table 2 with some depicted as figures (Fig. 4 and Fig. S1 and Fig. S2 in supporting information).

**Table 2:** Relationships among hormones both within and across tissue types.

	F_df_		P	R^2^		slope b			F_df_	P	R^2^	slope b
**All females across treatments**							**Alpha 24** **h**					
Muscle KT by Muscle T	F_1,66_ = 411.8		P<0.001	0.86		0.006	Muscle KT by Gonad KT		F_1,6_ = 28.4	P = 0.002	0.83	0.132
Gonad KT by Gonad T	F_1,65_ = 22.7		P<0.001	0.26		0.005	**Beta 24** **h**					
Gonad KT by Muscle T	F_1,65_ = 5.4		P = 0.023	0.08		−0.006	Gonad KT by Gonad T		F_1,6_ = 2976.1	P<0.0001	0.99	0.014
Gonad KT by Brain KT	F_1,65_ = 3.9		P = 0.054	0.06		0.073	Brain KT by Brain T		F_1,7_ = 152.6	P<0.0001	0.96	0.062
**Male stable**							Muscle KT by Muscle T		F_1,7_ = 82.5	P<0.0001	0.92	0.011
Muscle KT by Muscle T	F_1,8_ = 95.2		P<0.0001	0.92		0.006	Gonad E_2_ by Brain E_2_		F_1,7_ = 7.8	P = 0.026	0.53	0.037
**Alpha stable**							**Alpha 6d**					
Gonad KT by Gonad T	F_1,8_ = 200.6		P<0.0001	0.96		0.013	Gonad E_2_ by Brain E_2_		F_1,4_ = 18.2	P = 0.013	0.82	10.40
Muscle KT by Muscle T	F_1,8_ = 87.4		P<0.0001	0.92		0.005	Muscle T by Brain E_2_		F_1,6_ = 17.8	P = 0.006	0.75	0.238
Gonad E_2_ by Muscle E_2_	F_1,7_ = 6.2		P = 0.042	0.47		−14.64	Gonad KT by Brain E_2_		F_1,6_ = 12.8	P = 0.012	0.68	−6.400
**Beta stable**							Gonad E_2_ by Gonad KT		F_1,6_ = 11.8	P = 0.014	0.66	−85.00
Muscle KT by Muscle T	F_1,8_ = 305.4		P<0.0001	0.97		0.005	Gonad KT by Muscle T		F_1,8_ = 10.0	P = 0.013	0.56	−1.589
Gonad KT by Gonad T	F_1,8_ = 77.00		P<0.0001	0.91		0.010	**Beta 6d**					
**Gamma stable**							Muscle KT by Muscle T		F_1,8_ = 203.8	P<0.0001	0.96	0.006
Gonad KT by Gonad T	F_1,8_ = 184.8		P<0.0001	0.96		0.007	Brain KT by Brain T		F_1,8_ = 38.4	P = 0.0003	0.83	0.086
Muscle KT by Muscle T	F_1,6_ = 23.5		P = 0.003	0.80		0.006	Muscle KT by Gonad KT		F_1,8_ = 14.4	P = 0.005	0.64	0.383
Gonad KT by Brain T	F_1,8_ = 11.1		P = 0.011	0.58		0.001	Gonad KT by Muscle T		F_1,8_ = 8.2	P = 0.021	0.51	54.92
Gonad T by Brain T	F_1,8_ = 5.7		P = 0.044	0.42		0.088	Gonad KT by Gonad T		F_1,8_ = 7.7	P = 0.024	0.49	0.012

All fish together	F_df_	P	R^2^	slope b								
Muscle KT by Muscle T	F_1,75_ = 456.3	P<0.0001	0.86	0.006								
Gonad KT by Gonad T	F_1,75_ = 24.7	P<0.0001	0.25	0.004								
Muscle E_2_ by Brain E_2_	F_1,72_ = 9.9	P = 0.002	0.12	0.197								
Gonad KT by Brain KT	F_1,72_ = 5.3	P = 0.024	0.07	0.075								

Within each section (e.g., ‘Male stable’), the results are arranged in order of decreasing significance; regression statistics are provided.

#### Among-tissue relationships for a given hormone

First, we compared levels of the same hormone in different tissues to test whether changes occur across all tissues in a uniform way. When all fish were included in the analysis, there was a significant positive relationship between gonad KT and brain KT (Fig. S1A) but it was not significant when considering each class of fish separately except for gammas in stable groups. Testosterone in the brain and gonad were also significantly positively related only for gamma females in stable groups. Muscle KT and gonad KT were significantly positively related for betas rising to the alpha position at 6d and sex-changing alphas at 24 h following male removal (Fig. S1D).

Muscle and brain E_2_ concentrations were significantly positively related at the population level but this trend did not hold for any of the individual status classes. There was a significant positive relationship between gonad and brain E_2_ in betas rising to the alpha position at 24 h (Fig. S1B) and sex-changing alphas at 6d following male removal (Fig. S1C).

#### Within-tissue relationships among hormones

Second, we analyzed whether there was any relationship between different hormones in the same tissue. There were highly significant positive relationships between muscle KT and muscle T (Fig. 4A) as well as gonad KT and gonad T (Fig. 4B); these relationships were very consistent across the sexes, status classes, and time points. Only sex-changing alphas at 24 h and 6d following male removal failed to show relationships between the two androgens in gonad and muscle (Fig. 4C–E). Gonad E_2_ and KT concentrations were significantly negatively related only for sex-changing alphas at 6d following male removal (Fig. 4F). Brain KT and T concentrations were significantly positively related only in betas rising to the alpha position at 24 h and 6d following male removal (Fig. S2A–B).

### Hormone by Behavior Correlations

**Figure 5:**
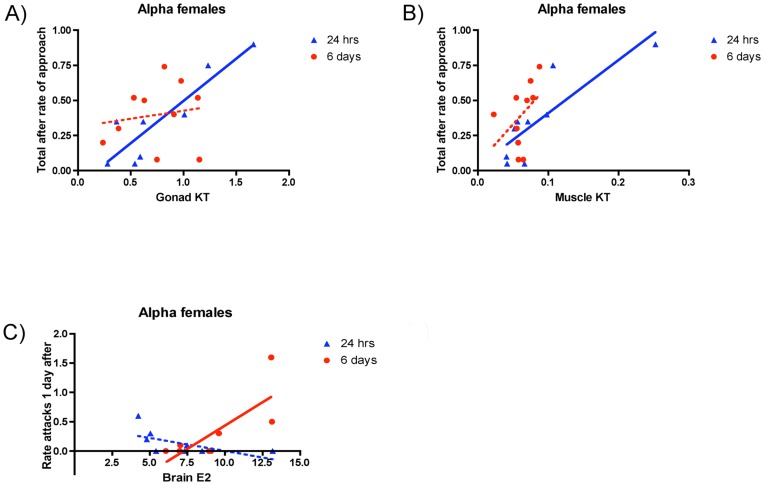
Correlations between hormones and behavior. Correlation between the total rate of approaches performed after male removal and concentration of KT in the gonad (A), and in the muscle (B); and between the rate of attacks performed the day after male removal and concentration of E_2_ in the brain (C). The values refer to alpha females in sex changing groups collected 24 hours and 6 days after male removal. A solid line means that the associated p value is significant (p<0.05) while dotted lines refer to non-significant correlations.

We examined the relationships between steroid concentrations in each tissue type and the behavior (approaches, attacks, displacements given, courtship, threat displays and displacement received) of animals in both stable groups and after male removal. All results described in this section refer to Table 3 with some depicted as figures (Fig. 5 and Fig. S3). With few exceptions, all hormone-behavior relationships occurred in sex-changing females and betas rising to the alpha position.

**Table 3:** Summary of hormone-behavior relationships.

	*Tissue and* *Steroid*	*Behavior*	F_df_	P	R^2^	slope b
**Male, stable 7d**	Gonad E_2_	Total displacements given	F_1,8_ = 9.8	P = 0.014	0.55	−6.54
	Gonad E_2_	Total approaches+aggression	F_1,8_ = 8.0	P = 0.022	0.50	−2.82
	Gonad E_2_	Total approaches	F_1,8_ = 6.9	P = 0.031	0.46	−5.49
**Sex-changing female, 24** **h**	Muscle KT	Total approaches before+after male removal	F_1,7_ = 108.4	P<0.0001	0.94	0.19
	Gonad KT	Total approaches before+after male removal	F_1,6_ = 25.9	P = 0.002	0.81	1.20
	Gonad KT	Total approaches after male removal	F_1,6_ = 25.0	P = 0.003	0.81	1.35
	Muscle KT	Total approaches after male removal	F_1,7_ = 17.1	P = 0.004	0.71	0.19
	Gonad KT	Approaches 1 h after male removal	F_1,6_ = 13.1	P = 0.011	0.69	1.01
	Muscle KT	Approaches 1 h after male removal	F_1,7_ = 5.9	P = 0.046	0.46	0.12
**Sex-changing female, 6d**	Muscle E_2_	Attacks 1 h after male removal	F_1,8_ = 16.8	P = 0.004	0.68	1.58
	Muscle E_2_	Total approaches+aggression 1 h after male removal	F_1,8_ = 16.7	P = 0.004	0.68	0.53
	Muscle E_2_	Displacements given 1 h after male removal	F_1,8_ = 15.5	P = 0.004	0.66	1.11
	Gonad KT	Total approaches before male removal	F_1,8_ = 14.2	P = 0.006	0.64	−1.66
	Brain E_2_	Attacks 1d after male removal	F_1,6_ = 8.9	P = 0.025	0.60	3.75
	Muscle E_2_	Approaches 1 h after male removal	F_1,8_ = 9.7	P = 0.014	0.55	2.27
	Muscle KT	Total displacements given after male removal	F_1,8_ = 5.8	P = 0.042	0.42	0.03
**Beta rising to alpha, 24** **h**	Brain KT	Times displaced 1d after male removal	F_1,7_ = 14.7	P = 0.007	0.68	3.02
	Brain T	Times displaced 1d after male removal	F_1,7_ = 9.5	P = 0.018	0.58	44.24
**Beta rising to alpha, 6d**	Gonad KT	Total times displaced after male removal	F_1,8_ = 18.5	P = 0.003	0.70	0.39
	Gonad E_2_	Total approaches+aggression beforeand after male removal	F_1,6_ = 13.5	P = 0.010	0.69	540.60
	Gonad E_2_	Total displacements given before male removal	F_1,6_ = 10.6	P = 0.017	0.64	495.95
	Brain T	Total times displaced 5d (PM) after male removal	F_1,8_ = 10.67	P = 0.011	0.57	−33.84
	Brain KT	Total times displaced 5d (PM) after male removal	F_1,8_ = 10.0	P = 0.013	0.56	−3.15
	Gonad T	Total approaches+aggression 5d(PM) after male removal	F_1,8_ = 5.6	P = 0.046	0.41	−11.73
	Gonad T	Displacements given 5d (PM) after male removal	F_1,8_ = 5.6	P = 0.046	0.41	−23.46
	Gonad KT	Approaches 5d (PM) after male removal	F_1,8_ = 5.5	P = 0.047	0.41	−0.44

Within each section (e.g., ‘Male, stable 7d’), the results are arranged in order of decreasing significance; regression statistics are provided.

#### Stable groups

Males of stable groups that were more aggressive (e.g., approaches, displacements given) had significantly less gonad E_2_. Aside from this, there were no significant relationships between tissue hormones and the behavior of animals in stable groups.

#### Sex-changing females

There was a significant positive relationship between both muscle and gonad (but not brain) KT of sex-changing females 24 h after male removal and the total number of approaches delivered to subordinate group members in the first day (Fig. 5A–B) as well as the number of approaches delivered in the first hour after male removal. Brain E_2_ of sex-changing females 6d after male removal also was significantly positively related to attacks delivered on the first day following male removal (Fig. 5C).

#### Betas rising

Brain T and KT of betas rising to the alpha position 24 h after male removal were significantly positively related to the number of times they were displaced by the sex-changing female following male removal (Fig. S3A–B). Brain T and KT of betas 6d after male removal instead showed a significant negative relationship with the rate of displacements received 5 days after male removal (Fig. S3C–D).

## Discussion

Our results demonstrate that levels of steroid hormones are altered during sex change but that these alterations do not follow the same pattern in all tissues. In addition, the strong positive relationship between androgens within different tissues is lost during sex change.

We also found a sex difference for T and E_2_ but not for KT. The higher E_2_ levels we found in female tissue are consistent with previous work on *L. dalli* that measured levels of steroids from water samples [39], and with the higher aromatase activity in females relative to males [17]. While water samples did not identify any sex difference in T nor KT [39], the present study shows that females have higher T than males in the gonads. Since brain levels of T are much higher than gonad levels and are not sexually dimorphic, they might mask the gonadal sex difference when using methods that average over the whole body (e.g., plasma or water-borne samples). Also, the very low levels of KT detected in *L. dalli* suggest that the lack of sex difference in KT is due to low levels in males rather than unusually high levels in females. These results are different from many fish species where males have much higher KT levels than females [41], but similar to what was found in another bidirectional hermaphrodite, *Gobiodon histrio,* where whole body concentrations of KT were below detection limits in both males and females [30]. 11-ketotestosterone is often viewed as the key hormone for regulating male-typical morphology in teleost fishes [41] and the lack of sex differences in KT in sequential bi-directional sex changers (like *L. dalli* and *G. histrio*) is consistent with their reduced overall sexual dimorphism and with the hypothesis that these fish reduce KT production across their life history so as to maintain the capacity for adult sex change in both directions.

### Different Tissues Present Different Concentrations of Steroids

During sex change, different tissues responded independently to the same social manipulation, demonstrating that tissues can be autonomous when it comes to steroid synthesis, binding, or uptake in response to changes in the social environment. We were surprised to find very few significant correlations when comparing the same hormone across different tissues within individuals. One might expect that if steroids are completely free to reach all organs through circulation, then there should be a positive correlation in steroid levels across tissues within individuals. For example, fish whose gonad is producing high levels of E_2_ would also have more E_2_ in brain and/or muscle. Instead, our results indicate that each tissue is remarkably independent with regard to steroid concentrations. This result challenges the central dogma that steroids produced in the gonad represent ‘the’ whole body regulator of steroid actions, and could be viewed as a broad application of the law of mass action, which suggests that the concentration and affinity of receptors, binding globulins, and synthetic/degradative enzymes determines the functional capacity of a signaling biochemical in a given tissue. Future studies must consider all these factors together in a tissue to fully understand the mechanism of action of steroid hormones.

The results for androgens were unexpected; both T and KT were much higher in the brain than in gonad and muscle. The most likely explanation is that the brain is an important site for androgen synthesis. There is evidence that fish brain homogenates can produce T, E_2_ and KT [12], [13], [15], [42]. An alternative explanation is that androgens are sequestered from circulation and accumulated in the brain by androgen receptors and/or steroid binding globulins (SBG) at a concentration higher than the gonadal source. Steroid binding globulins can transport steroid hormones from circulation into cells [43], and can be internalized by neurons in rats [44]. Also, different rates of steroid degradation associated with each specific tissue could explain the different levels of T and KT that we observed among tissue types.

The only strong hormone correlation that held true for all females in stable groups was between KT and T in both muscle and gonad. Similarly, in male *Tilapia zillii*, plasma KT was positively correlated with plasma T [45]. An obvious explanation is that T is a precursor of KT but for the same reason, we would expect to see the same correlation also in the brain. 11-ketotestosterone might not be directly synthesized in the brain or its rate of synthesis could be independently and locally regulated such that product and substrate are not tightly coupled. Also, KT could be binding SBG in the brain with a different affinity than T [46], [47], [48] affecting its metabolism and precluding direct relationships with its precursor.

We found no significant difference in T between gonad and muscle. Testosterone present in the muscle could be coming through circulation or could be produced by muscle tissue but there is no evidence for any 17β-HSD activity in muscle of male rainbow trout [15].

Because we found correlations between KT and its precursor T, we also expected correlations between T and another of its metabolites, E_2_. In the cichlid *N. pulcher* T correlated with both E_2_ and KT in plasma [10]. On the other hand, in the protandrous *Amphiprion melanopus* T correlated with E_2_ in females but not in males, and none of the correlations were present in fish undergoing sex change [49]. In *L. dalli,* we did not find a correlation between T and E_2_ in any of the tissues, which suggests that E_2_ could be synthesized from estrone after aromatization of androstenedione as in the protogynous wrasse *Pseudolabrus sieboldi* [36]. Further work is necessary to determine whether *L. dalli* employs this alternative pathway.

### Steroid Responses During Sex Change

After removal of the male from the social groups, we observed changes in steroid levels in tissues of sex-changing females supporting a critical role of KT in the regulation of sex change. Comparing the steroid response of alpha females with beta females rising to the alpha position allows one to partition hormonal responses due to changes in social status from those due to sex change. Specifically, KT in alphas increases at 24 h and 6d relative to alphas in stable groups, while it does not change significantly in any tissue of betas rising to alphas. These results demonstrate that changes in KT are closely associated with sex change, rather than a change in social status following male removal. In *L. dalli*, KT can induce masculinization of the genital papilla [50], so the increase is consistent with the male typical papilla ratio we found in sex changing alpha females at 6 days. The rise in KT during sex change might be needed to induce spermatogenesis or the onset of male typical behavior, but is not necessary for the maintenance of male function, as indicated by the lack of sex differences in KT in stable groups. In stoplight parrotfish, serum KT increased in females during sex change, and then again when changing color into the terminal male phase [33] supporting the hypothesis that KT is required during transitions from one reproductive state to another. The mechanism of action of KT is not clear yet, but administration of KT induced the start of sex change and depressed levels of E_2_ in stoplight parrotfish [51], and in the honeycomb grouper *Epinephelus merra* [32]. Increased KT during sex change might function primarily to inhibit E_2_ production allowing oocytes to regress and gonadal sex change to proceed.

We found increased KT in both brain and gonads of sex changing alpha females, so extra-gonadal synthesis of KT could be essential to initiate sex change. Interestingly, in stable groups alpha females had the lowest levels of KT in all tissues and this might make the rapid increase in brain and gonadal KT that happens during sex change even more dramatic and effective.

In *L. dalli*, aromatase activity decreases during the first stages of sex change in the brain but not in the gonad [17] so we expected a drop in brain E_2_ levels during sex change, however this was not the case. Changes in E_2_ levels do not seem to play a major role in the initiation of sex change. In fact, sex changing alphas 6 days after male removal still have E_2_ levels similar to alpha females in stable groups and much higher than males. Our results show that the ovary in *L. dalli* has the highest levels of E_2_ while in other teleosts aromatase activity is typically reported to be higher in brain than gonad [52], [13] *L. dalli* brain has very high level of T, which is the substrate for aromatase, so we would expect much higher levels of E_2_ in the brain than in the gonad if aromatase activity was equivalent.

Since in *L. dalli* the gonad produces much more E_2_ than the brain, gonadal E_2_ coming into the brain from circulation could swamp our ability to detect differences in brain E_2_ levels during the early phases of sex change, or brain aromatase and E_2_ could just be modulated at a very fine local scale, consistent with a synaptocrine regulation of E_2_ in the brain [53].

In contrast with what happens to KT, concentrations of T in *L. dalli* decrease in brain and muscle of both alpha and beta females after male removal, suggesting an association of T with the change in social circumstance rather than the induction of sex change *per se*. Other animals have been shown to change steroid levels after being presented with an individual of the opposite sex [54], [55], [56]. The presence of males might increase T levels in female *L. dalli* in stable groups while male removal might precipitate a decrease in T synthesis. It is worth noting that the strong relationship between KT and T in the other females was lacking in alphas undergoing sex change (Fig. 4). Both alphas and betas do show the drop in T brain levels in response to male removal suggesting that the increase in KT which is only present in alphas, is responsible of the lack of correlation between T and KT. Further work will be needed to understand the mechanism peculiar to the sex changing fish that can induce this rapid increase in KT.

### Androgens and Aggression

We found a positive relationship between androgens and aggression in alpha females but only after male removal when the social situation became unstable. For sex changing females at 24 h, gonad and muscle KT positively correlated with the total rate of approaches to other fish, and muscle KT 6d after male removal with the rate of displacement of subordinate females. We found that alpha female E_2_ levels correlated with aggression 6d after male removal. The time shortly after male removal is the most critical phase in the onset of sex change because that is when the dominant female takes the place of the male in the hierarchy. Thus, aggression levels displayed by an alpha female at that stage might affect hormone levels on the following days. On the day of male removal, *L. dalli* sex changing females often show increased aggression (e.g., [26], [17]) that can last for several days, but then returns to levels similar to stable groups. Most of the significant correlations we found involve the behavior after male removal, so sex steroids might affect behavior only in situations of social instability [4] as suggested by the “challenge hypothesis” [5]. The vast majority of studies finding a relationship between androgens and aggression use the resident/intruder paradigm, while in our experiment the fish already established a stable social structure so the hormone-behavior responses might be less dramatic.

### Conclusions

Our results show that different tissues within the same animal often have very different steroid levels, and these tissues can respond differently to the same social stimulus. Thus, measures of circulating levels of steroids may not provide adequate information to describe the local steroidal milieu and thus the primary effects of steroid action, which take place at the local level. Our data suggest that the gonad in *L. dalli* is the main source of estrogen and that the brain is an important source of androgens. Future studies should seek to determine whether these tissue differences are due to differential rates of synthesis/catabolism or active compartmentalization by steroid binding globulins or steroid receptors. Androgens seem to be an important candidate for steroids that translate changes in the social environment into changes in sexual phenotype. In fact, during the first 6 days of sex change, E_2_ levels stay stable in all tissues, T decreases dramatically in brain and muscle, and KT increases in the brain. The drop in T could act as a chemical message to signal the lack of mating opportunity, but it is not sufficient to induce sex change without the increase in KT, which only occurs in the sex changing alpha female that is now the new dominant animal. We propose that an increase in brain KT and an associated drop in brain T may serve as a steroidal switch that initiates sex change. Future studies also should consider regional differences in brain steroid levels, and the potential for pulsatile steroid secretion and neurosteroids other than those measured in this study (e.g. DHEA-S) to mediate the behavioral and physiological responses to changes in status and sex. Socially regulated sex change provides an excellent model in which to study the evolution of novel mechanisms for allowing social interactions to regulate steroid signaling and reproductive phenotype.

## Supporting Information

Figure S1 **Steroid correlations across tissues.** Correlations between concentration of KT in the gonad versus KT in the brain (A) across all fish from stable and sex changing groups, between concentrations of E_2_ in the gonad versus the brain of beta (B) and alpha (C) females, and between concentration of KT in the muscle versus the gonad of alpha females (D). A solid line means that the associated p value is significant (p<0.05) while dotted lines are associated with non-significant correlations.
(DOC)

Figure S2 **Correlations across hormones.** Correlation between concentration of T versus KT in the brain of beta females in stable groups (A), and in sex changing groups (B). Correlation between concentration of E_2_ in the brain versus KT in the gonad (C) of alpha females. A solid line means that the associated p value is significant (p<0.05) while dotted lines refer to non-significant correlations.
(DOC)

Figure S3 **Correlation between hormones and behavior.** Correlation between the rate of displacements received 1 day after male removal and brain concentration of T (A), and KT (B); between the rate of displacements received in the afternoon 5 days after male removal and brain concentration of T (C) and KT (D); between the total rate of displacements received after male removal and concentration of KT in the gonad (E); and between the rate of displacements given before male removal and concentration of E_2_ in the gonad (F). The values are from beta females in sex changing groups collected 24 hours and 6 days after male removal. A solid line means that the associated p value is significant (p<0.05) while dotted lines refer to non-significant correlations.
(DOC)

Table S1 List of all linear contrasts that are significant or show a trend towards significant values for fish in stable groups.
(DOC)

Table S2 List of all linear contrasts that are significant or show a trend towards significant values for sex changing groups. These linear contrasts compare alpha and beta females in stable groups and in groups undergoing sex change (24 hours and 6 days).
(DOC)

Table S3 Three-way linear contrasts comparing females.
(DOC)

Table S4 Linear contrasts between stable males and females in sex changing groups.
(DOC)

Table S5 Three-way linear contrasts comparing males and females.
(DOC)

Methods S1 (DOC)
