# Supplementary figures and images for: Differential Responses of Brain, Gonad and Muscle Steroid Levels to Changes in Social Status and Sex in a Sequential and Bidirectional Hermaphroditic Fish
Source: PLoS One. 2012 Dec 10;7(12):e51158. doi: 10.1371/journal.pone.0051158 (PMC3519529; doi:10.1371/journal.pone.0051158)

**Figure S1:**


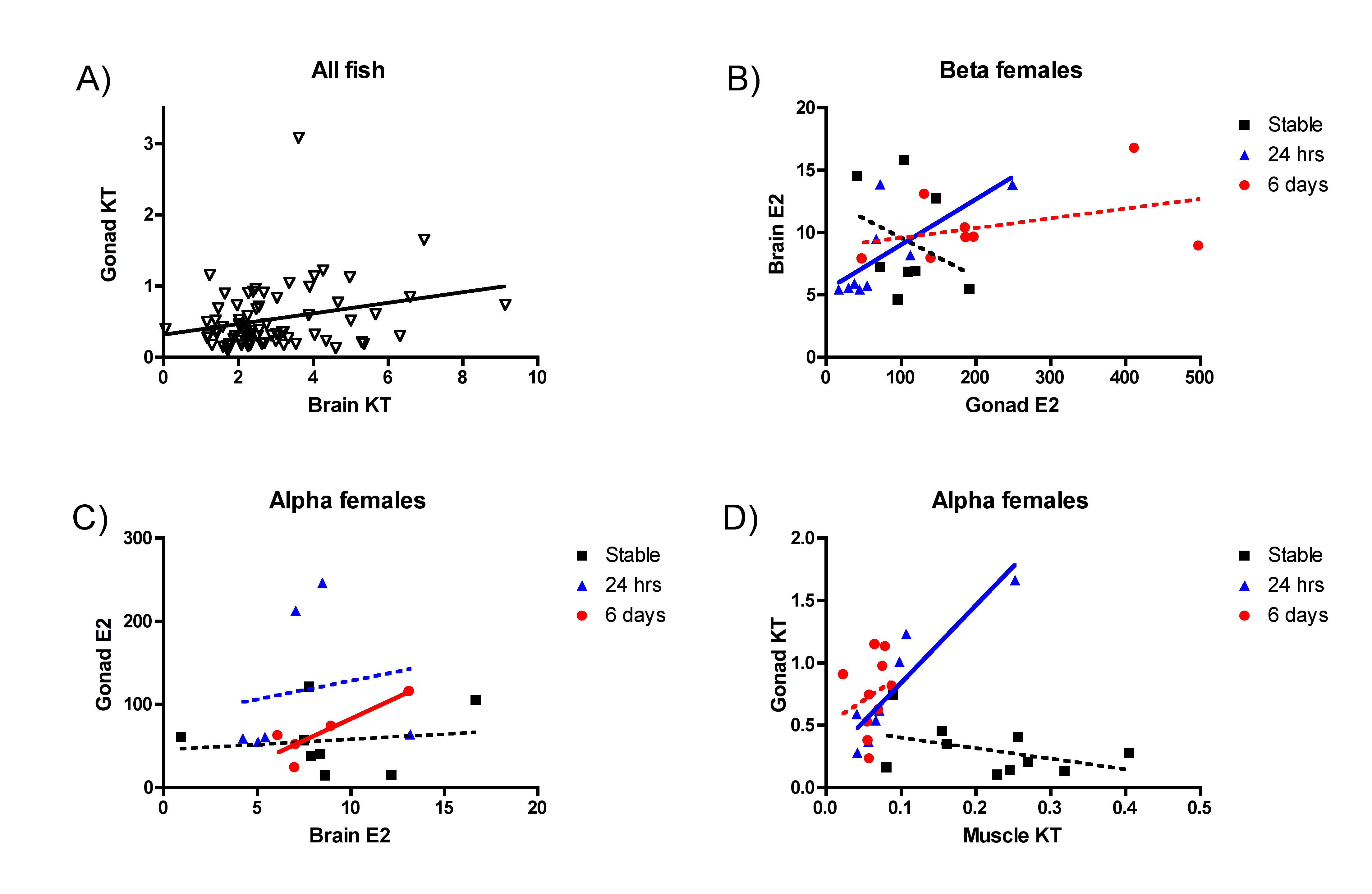

Supplement: Figure S1 — Steroid correlations across tissues. Correlations between concentration of KT in the gonad versus KT in the brain (A) across all fish from stable and sex changing groups, between concentrations of E2 in the gonad versus the brain of beta (B) and alpha (C) females, and between concentration of KT in the muscle versus the gonad of alpha females (D). A solid line means that the associated p value is significant (p<0.05) while dotted lines are associated with non-significant correlations. (DOC) [file pone.0051158.s001.doc]

**Figure S2:**


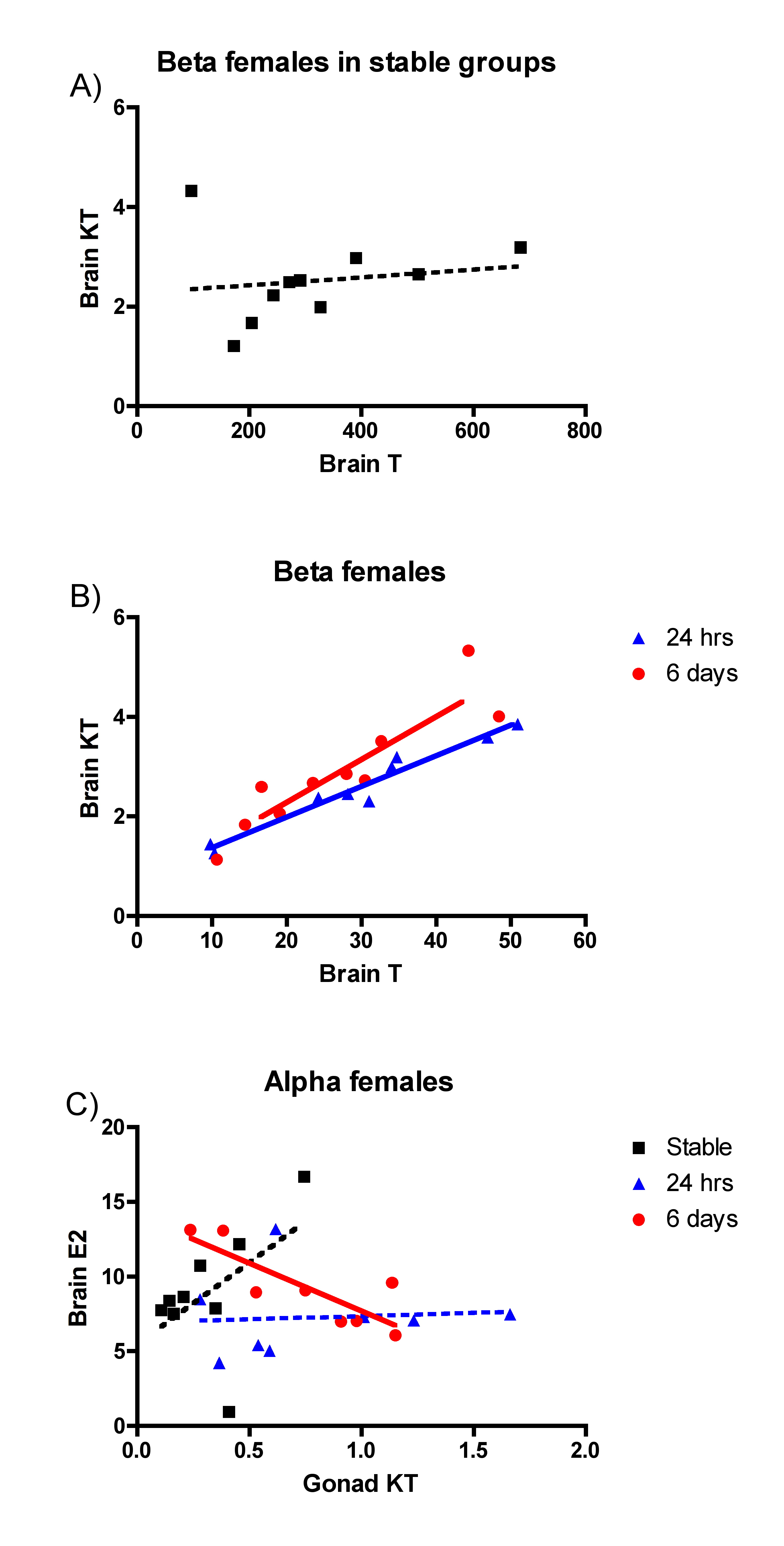

Supplement: Figure S2 — Correlations across hormones. Correlation between concentration of T versus KT in the brain of beta females in stable groups (A), and in sex changing groups (B). Correlation between concentration of E2 in the brain versus KT in the gonad (C) of alpha females. A solid line means that the associated p value is significant (p<0.05) while dotted lines refer to non-significant correlations. (DOC) [file pone.0051158.s002.doc]

**Figure S3:**


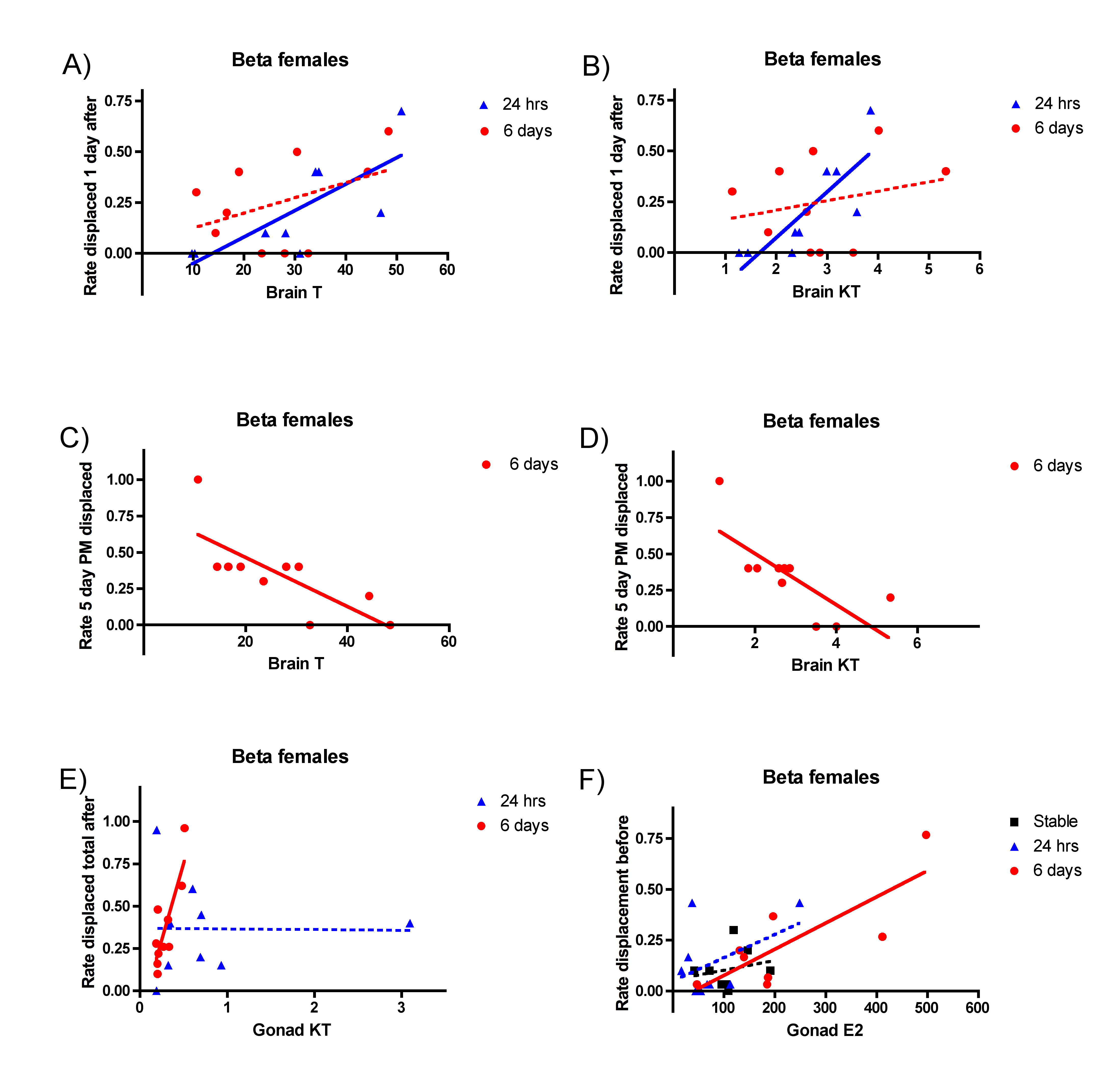

Supplement: Figure S3 — Correlation between hormones and behavior. Correlation between the rate of displacements received 1 day after male removal and brain concentration of T (A), and KT (B); between the rate of displacements received in the afternoon 5 days after male removal and brain concentration of T (C) and KT (D); between the total rate of displacements received after male removal and concentration of KT in the gonad (E); and between the rate of displacements given before male removal and concentration of E2 in the gonad (F). The values are from beta females in sex changing groups collected 24 hours and 6 days after male removal. A solid line means that the associated p value is significant (p<0.05) while dotted lines refer to non-significant correlations. (DOC) [file pone.0051158.s003.doc]
