# Supplementary material for: Differential Responses of Brain, Gonad and Muscle Steroid Levels to Changes in Social Status and Sex in a Sequential and Bidirectional Hermaphroditic Fish
Source: PLoS One. 2012 Dec 10;7(12):e51158. doi: 10.1371/journal.pone.0051158 (PMC3519529; doi:10.1371/journal.pone.0051158)
Supplement: Table S1 — List of all linear contrasts that are significant or show a trend towards significant values for fish in stable groups. (DOC) [file pone.0051158.s004.doc]

**Table S1:** List of all linear contrasts that are significant or show a trend towards significant values for fish in stable groups.

| **Contrast** | **p-value, F ratio** |
| --- | --- |
| ***Estradiol*** |  |
| Female gonad > male gonad | p<0.0001, F_1,104_=202.95 |
| Beta gonad > beta muscle | p<0.0001, F_1,104_=187.52 |
| Beta gonad > male gonad | p<0.0001, F_1,104_=175.72 |
| Gamma gonad > gamma muscle | p<0.0001, F_1,104_=145.48 |
| Gamma gonad > male gonad | p<0.0001, F_1,104_=141.91 |
| Beta gonad > beta brain | p<0.0001, F_1,104_=100.63 |
| Alpha gonad > alpha muscle | p<0.0001, F_1,104_=94.63 |
| Alpha gonad > male gonad | p<0.0001, F_1,104_=94.61 |
| Gamma gonad > gamma brain | p<0.0001, F_1,104_=76.98 |
| Female muscle > male muscle | p<0.0001, F_1,104_=50.99 |
| Alpha gonad > alpha brain | p<0.0001, F_1,104_=45.70 |
| Male brain > male muscle | p<0.0001, F_1,104_=42.54 |
| Male gonad > male muscle | p<0.0001, F_1,104_=36.32 |
| Alpha muscle > male muscle | p<0.0001, F_1,104_=36.31 |
| Gamma muscle > male muscle | p<0.0001, F_1,104_=34.54 |
| Beta muscle > male muscle | p<0.0001, F_1,104_=31.24 |
| Beta brain > beta muscle | p=0.0001, F_1,104_=13.41 |
| Gamma brain > gamma muscle | p=0.0005, F_1,104_=10.81 |
| Female brain > male brain | p=0.0009, F_1,104_=9.69 |
| Beta brain > male brain | p=0.0033, F_1,104_=7.45 |
| Gamma gonad > alpha gonad | p=0.0035, F_1,104_=4.78 |
| Alpha brain > alpha muscle | p=0.0036, F_1,104_=7.33 |
| Gamma brain > male brain | p=0.0044, F_1,104_=6.99 |

**Table S1:** List of all linear contrasts that are significant or show a trend towards significant values for fish in stable groups (continued).

| Beta gonad > alpha gonad | p=0.0051, F_1,104_=12.45 |
| --- | --- |
| Alpha brain > male brain | p=0.0160, F_1,104_=4.95 |
| ***Testosterone*** |  |
| Male brain > male gonad | p<0.0001, F_1,105_=94.16 |
| Male brain > male muscle | p<0.0001, F_1,105_=60.47 |
| Alpha brain > alpha gonad | p<0.0001, F_1,105_=52.37 |
| Beta brain > beta gonad | p<0.0001, F_1,105_=58.26 |
| Gamma brain > gamma gonad | p<0.0001, F_1,105_=51.82 |
| Beta brain > beta muscle | p<0.0001, F_1,105_=47.44 |
| Gamma brain > gamma muscle | p<0.0001, F_1,105_=42.83 |
| Alpha brain > alpha muscle | p<0.0001, F_1,105_=39.79 |
| Gamma gonad > male gonad | p=0.0006, F_1,105_=12.60 |
| Female gonad > male gonad | p=0.0012, F_1,105_=11.11 |
| Beta gonad > male gonad | p=0.0045, F_1,105_=8.43 |
| Gamma muscle > male muscle | p=0.0438, F_1,105_=4.16 |
| Gamma gonad > alpha gonad | p=0.0690, F_1,105_=3.38 |
| ***11-Ketotestosterone*** |  |
| Gamma brain > beta muscle | p<0.0001, F_1,106_=100.73 |
| Gamma brain > beta gonad | p<0.0001, F_1,106_=92.19 |
| Beta brain > beta muscle | p<0.0001, F_1,106_=78.57 |
| Male brain > male muscle | p<0.0001, F_1,106_=67.16 |
| Beta brain > beta gonad | p<0.0001, F_1,106_=60.95 |
| Male brain > male gonad | p<0.0001, F_1,106_=41.42 |
| Alpha brain > alpha muscle | p<0.0001, F_1,106_=41.06 |
| Alpha brain > alpha gonad | p<0.0001, F_1,106_=34.62 |
| Gamma Brain > alpha Brain | p<0.0001, F_1,106_=19.77 |

**Table S1:** List of all linear contrasts that are significant or show a trend towards significant values for fish in stable groups (continued).

| Beta Brain > alpha Brain | p=0.0100, F_1,106_=6.88 |
| --- | --- |
| Gamma Brain > male Brain | p=0.0115, F_1,106_=6.61 |
| Male brain > alpha brain | p=0.0547, F_1,106_=3.77 |
| Gamma brain > beta brain | p=0.0638, F_1,106_=3.51 |
