# Supplementary material for: Differential Responses of Brain, Gonad and Muscle Steroid Levels to Changes in Social Status and Sex in a Sequential and Bidirectional Hermaphroditic Fish
Source: PLoS One. 2012 Dec 10;7(12):e51158. doi: 10.1371/journal.pone.0051158 (PMC3519529; doi:10.1371/journal.pone.0051158)
Supplement: Table S2 — List of all linear contrasts that are significant or show a trend towards significant values for sex changing groups. These linear contrasts compare alpha and beta females in stable groups and in groups undergoing sex change (24 hours and 6 days). (DOC) [file pone.0051158.s005.doc]

**Table S2:** List of all linear contrasts that are significant or show a trend towards significant values for sex changing groups. These linear contrasts compare alpha and beta females in stable groups and in groups undergoing sex change (24 hours and 6 days).

| **Contrast** | **p-value, F ratio** |
| --- | --- |
| ***Estradiol*** |  |
| Beta gonad > beta muscle | p<0.0001, F_1,152_=562.98 |
| Alpha gonad > alpha muscle | p<0.0001, F_1,152_=453.92 |
| 6d gonad > 6d muscle | p<0.0001, F_1,152_=414.25 |
| 24h gonad > 24h muscle | p<0.0001, F_1,152_=302.96 |
| Stable gonad > stable muscle | p<0.0001, F_1,152_=302.94 |
| Beta gonad > beta brain | p<0.0001, F_1,152_=280.95 |
| Alpha gonad > alpha brain | p<0.0001, F_1,152_=195.92 |
| 6d gonad > 6d brain | p<0.0001, F_1,152_=172.94 |
| 24h gonad > 24h brain | p<0.0001, F_1,152_=153.11 |
| Stable gonad > stable brain | p<0.0001, F_1,152_=144.94 |
| Alpha brain > alpha muscle | p<0.0001, F_1,152_=52.51 |
| Beta brain > beta muscle | p<0.0001, F_1,152_=52.42 |
| 6d brain > 6d muscle | p<0.0001, F_1,152_=49.57 |
| Stable brain > stable muscle | p<0.0001, F_1,152_=28.83 |
| 24h brain > 24h muscle | p<0.0001, F_1,152_=28.48 |
| Beta 6d > beta 24h | p=0.0001, F_1,152_=15.19 |
| Beta 6d > alpha 6d | p=0.0002, F_1,152_=14.27 |
| Beta gonad > alpha gonad | p=0.0053, F_1,152_=8.01 |
| 6d gonad > stable gonad | p=0.0100, F_1,152_=6.81 |
| Beta 6d > beta stable | p=0.0175, F_1,152_=5.78 |
| 6d gonad > 24h gonad | p=0.0361, F_1,152_=4.48 |
| Beta stable > alpha stable | p=0.0620, F_1,152_=3.54 |
| 6d brain > 24h brain | p=0.0694 F_1,152_=3.35 |

**Table S2:** List of all linear contrasts that are significant or show a trend towards significant values for sex changing groups (continued).

| ***Testosterone*** |  |
| --- | --- |
| Stable muscle > 6d muscle | p<0.0001, F_1,149_=211.39 |
| Beta brain > beta muscle | p<0.0001, F_1,149_=193.85 |
| Alpha brain > alpha muscle | p<0.0001, F_1,149_=190.88 |
| Stable muscle > 24h muscle | p<0.0001, F_1,149_=166.70 |
| 6d brain > 6d muscle | p<0.0001, F_1,149_=152.71 |
| Beta stable > beta 6d | p<0.0001, F_1,149_=142.35 |
| 24h brain > 24h muscle | p<0.0001, F_1,149_=141.47 |
| Stable brain > 6d brain | p<0.0001, F_1,149_=123.80 |
| Stable brain > stable gonad | p<0.0001, F_1,149_=119.64 |
| Alpha stable > alpha 6d | p<0.0001, F_1,149_=128.64 |
| 6d gonad > 6d muscle | p<0.0001, F_1,149_=110.97 |
| Beta stable > beta 24h | p<0.0001, F_1,149_=103.77 |
| Stable brain > 24h brain | p<0.0001, F_1,149_=102.92 |
| Stable brain > stable muscle | p<0.0001, F_1,149_=94.11 |
| Alpha stable > alpha 24h | p<0.0001, F_1,149_=90.54 |
| 24h gonad > 24h muscle | p<0.0001, F_1,149_=90.44 |
| Beta gonad > beta muscle | p<0.0001, F_1,149_=77.73 |
| Alpha brain > alpha gonad | p<0.0001, F_1,149_=48.54 |
| Alpha gonad > alpha muscle | p<0.0001, F_1,149_=45.95 |
| Beta brain > beta gonad | p<0.0001, F_1,149_=24.54 |
| Beta gonad > alpha gonad | p=0.0021, F_1,149_=9.76 |
| Stable gonad > 6d gonad | p=0.0071, F_1,149_=7.45 |
| 6d brain > 6d gonad | p=0.0342, F_1,149_=4.57 |
| Beta stable > alpha stable | p=0.0542, F_1,149_=3.77 |

**Table S2:** List of all linear contrasts that are significant or show a trend towards significant values for sex changing groups (continued).

| ***11-Ketotestosterone*** | |
| --- | --- |
| Alpha brain > alpha muscle | p<0.0001, F_1,152_=330.16 |
| Beta brain > beta muscle | p<0.0001, F_1,152_=323.96 |
| 6d brain > 6d muscle | p<0.0001, F_1,152_=303.06 |
| 24h brain > 24h muscle | p<0.0001, F_1,152_=266.77 |
| Beta brain > beta gonad | p<0.0001, F_1,152_=172.82 |
| Alpha brain > alpha gonad | p<0.0001, F_1,152_=156.57 |
| 6d brain > 6d gonad | p<0.0001, F_1,152_=149.88 |
| Stable brain > stable muscle | p<0.0001, F_1,152_=109.29 |
| 24h brain > 24h gonad | p<0.0001, F_1,152_=96.10 |
| Stable brain > stable gonad | p<0.0001, F_1,152_=87.89 |
| 24h gonad > 24h muscle | p<0.0001, F_1,152_=39.44 |
| Alpha gonad > alpha muscle | p<0.0001, F_1,152_=31.60 |
| 6d gonad > 6d muscle | p<0.0001, F_1,152_=28.35 |
| Beta gonad > beta muscle | p<0.0001, F_1,152_=23.55 |
| 6d brain > stable brain | p<0.0001, F_1,152_=17.64 |
| 24h brain > stable brain | p=0.0003, F_1,152_=13.57 |
| Alpha 24h > alpha stable | p=0.0005, F_1,152_=12.80 |
| Alpha 6d > alpha stable | p=0.0005, F_1,152_=12.72 |
| Stable muscle > 6d muscle | p=0.0036, F_1,152_=8.76 |
| Alpha 6d > beta 6d | p=0.0055, F_1,152_=7.94 |
| 24h gonad > stable gonad | p=0.0072, F_1,152_=7.42 |
| Stable muscle > 24h muscle | p=0.0075, F_1,152_=7.34 |
| Beta stable > alpha stable | p=0.0263, F_1,152_=5.04 |
