# Supplementary material for: Differential Responses of Brain, Gonad and Muscle Steroid Levels to Changes in Social Status and Sex in a Sequential and Bidirectional Hermaphroditic Fish
Source: PLoS One. 2012 Dec 10;7(12):e51158. doi: 10.1371/journal.pone.0051158 (PMC3519529; doi:10.1371/journal.pone.0051158)
Supplement: Table S3 — Three-way linear contrasts comparing females. (DOC) [file pone.0051158.s006.doc]

**Table S3:** Three-way linear contrasts comparing females.

|  |  |  | Stable Groups | | | | | | 24h Groups | | | | | | 6d Groups | | | | | |
| --- | --- | --- | --- | --- | --- | --- | --- | --- | --- | --- | --- | --- | --- | --- | --- | --- | --- | --- | --- | --- |
|  |  |  | α | | | β | | | α | | | β | | | α | | | β | | |
|  |  |  | B | G | M | B | G | M | B | G | M | B | G | M | B | G | M | B | G | M |
| Stable | α | B |  |  |  |  |  |  |  |  |  |  |  |  |  |  |  |  |  |  |
|  |  | G | 51.7 <0.0001 |  |  |  |  |  |  |  |  |  |  |  |  |  |  |  |  |  |
|  |  | M | 10.46  0.002 | 112.6  <0.0001 |  |  |  |  |  |  |  |  |  |  |  |  |  |  |  |  |
|  | β | B | 0.266  0.607 |  |  |  |  |  |  |  |  |  |  |  |  |  |  |  |  |  |
|  |  | G |  | 9.658  0.002 |  | 96.6  <0.0001 |  |  |  |  |  |  |  |  |  |  |  |  |  |  |
|  |  | M |  |  | 0.272  0.603 | 19.1  <0.0001 | 194.7  <0.0001 |  |  |  |  |  |  |  |  |  |  |  |  |  |
| 24h | α | B | 0.217  0.642 |  |  | 0.986  0.322 |  |  |  |  |  |  |  |  |  |  |  |  |  |  |
|  |  | G |  | 6.698  0.011 |  |  | 0.073  0.787 |  | 89.0  <0.0001 |  |  |  |  |  |  |  |  |  |  |  |
|  |  | M |  |  | 1.625  0.204 |  |  | 0.588  0.444 | 15.4  0.0001 | 167.7  <0.0001 |  |  |  |  |  |  |  |  |  |  |
|  | β | B | 0.007  0.932 |  |  | 0.183  0.670 |  |  | 0.303  0.583 |  |  |  |  |  |  |  |  |  |  |  |
|  |  | G |  | 0.827  0.365 |  |  | 4.952  0.028 |  |  | 3.149  0.078 |  | 64.2  <0.0001 |  |  |  |  |  |  |  |  |
|  |  | M |  |  | 0.151  0.698 |  |  | 0.014  0.905 |  |  | 0.747  0.389 | 13.1  0.0004 | 135.3  <0.0001 |  |  |  |  |  |  |  |
| 6d | α | B | 0.501  0.480 |  |  | 0.051  0.822 |  |  | 1.344  0.248 |  |  | 0.390  0.533 |  |  |  |  |  |  |  |  |
|  |  | G |  | 2.168  0.143 |  |  | 2.526  0.114 |  |  | 1.442  0.232 |  |  | 0.348  0.556 |  | 56.6  <0.0001 |  |  |  |  |  |
|  |  | M |  |  | 2.521  0.115 |  |  | 1.136  0.288 |  |  | 0.073  0.787 |  |  | 1.339  0.249 | 28.7  <0.0001 | 176.4  <0.0001 |  |  |  |  |
|  | β | B | 2.325  0.130 |  |  | 1.075  0.301 |  |  | 4.009  0.047 |  |  | 2.065  0.153 |  |  | 0.565  0.453 |  |  |  |  |  |
|  |  | G |  | 28.95  <0.0001 |  |  | 4.879  0.029 |  |  | 5.362  0.022 |  |  | 20.234  <0.0001 |  |  | 14.426  0.0002 |  | 125.0  <0.0001 |  |  |
|  |  | M |  |  | 0.094  0.759 |  |  | 0.687  0.408 |  |  | 2.477  0.118 |  |  | 0.472  0.493 |  |  | 3.591  0.060 | 21.0  <0.0001 | 240.3  <0.0001 |  |

Estradiol showed a significant 3-way interaction so we performed 3-way linear contrasts. Within each cell of the table, the F-value is shown on top, p-value on bottom, all F-values have df=1, 143. Yellow cells indicate that the contrast is significant; blue cells indicate a marginal significance. Stable alpha = alpha female; stable beta = beta female; in 24 hours and 6 days groups: alpha = sex-changing female; beta = beta female rising to alpha status; B = brain, G = gonad, M = muscle
