# Supplementary material for: Differential Responses of Brain, Gonad and Muscle Steroid Levels to Changes in Social Status and Sex in a Sequential and Bidirectional Hermaphroditic Fish
Source: PLoS One. 2012 Dec 10;7(12):e51158. doi: 10.1371/journal.pone.0051158 (PMC3519529; doi:10.1371/journal.pone.0051158)
Supplement: Table S4 — Linear contrasts between stable males and females in sex changing groups. (DOC) [file pone.0051158.s007.doc]

**Table S4:** Linear contrasts between stable males and females in sex changing groups.

| **Contrast** | **p-value, F ratio** |
| --- | --- |
| ***Estradiol*** |  |
| Beta gonad > beta muscle | p<0.0001, F_1,145_=419.19 |
| 6d gonad > 6d muscle | p<0.0001, F_1,145_=364.47 |
| Alpha gonad > alpha muscle | p<0.0001, F_1,145_=334.49 |
| 24h gonad > 24h muscle | p<0.0001, F_1,145_=266.55 |
| Beta gonad > beta brain | p<0.0001, F_1,145_=204.34 |
| 6d gonad > 6d brain | p<0.0001, F_1,145_=152.16 |
| Stable gonad > stable muscle | p<0.0001, F_1,145_=140.24 |
| 24h gonad > 24h brain | p<0.0001, F_1,145_=134.72 |
| 6d gonad > stable gonad | p<0.0001, F_1,145_=128.45 |
| Alpha 6d > alpha stable | p<0.0001, F_1,145_=126.43 |
| Alpha 24h > alpha stable | p<0.0001, F_1,145_=124.53 |
| Beta stable > alpha stable | p<0.0001, F_1,145_=122.70 |
| Alpha gonad > alpha brain | p<0.0001, F_1,145_=86.69 |
| Alpha brain > alpha muscle | p<0.0001, F_1,145_=84.36 |
| 24h gonad > stable gonad | p<0.0001, F_1,145_=80.01 |
| Stable brain > stable muscle | p<0.0001, F_1,145_=52.88 |
| 6d brain > 6d muscle | p<0.0001, F_1,145_=43.61 |
| Beta gonad > alpha gonad | p<0.0001, F_1,145_=40.59 |
| Beta brain > beta muscle | p<0.0001, F_1,145_=38.16 |
| Beta muscle > alpha muscle | p<0.0001, F_1,145_=28.30 |

**Table S4:** Linear contrasts between stable males and females in sex changing groups (continued).

| 24h brain > 24h muscle | p<0.0001, F_1,145_=25.06 |
| --- | --- |
| Stable gonad > stable brain | p<0.0001, F_1,145_=20.34 |
| Beta 6d > beta stable | p<0.0001, F_1,145_=16.20 |
| 6d muscle > stable muscle | p=0.0001, F_1,145_=15.41 |
| Beta 6d > beta 24h | p=0.0004, F_1,145_=13.36 |
| 24h muscle > stable muscle | p=0.0005, F_1,145_=12.56 |
| Beta 6d > alpha 6d | p=0.0005, F_1,145_=12.55 |
| 6d brain > stable brain | p=0.0016, F_1,145_=10.36 |
| Beta brain > alpha brain | p=0.0353, F_1,145_=4.51 |
| 6d gonad > 24h gonad | p=0.0491, F_1,145_=3.94 |
| ***Testosterone*** |  |
| Alpha brain > alpha muscle | p<0.0001, F_1,149_=211.16 |
| Beta brain > beta muscle | p<0.0001, F_1,149_=178.87 |
| Stable muscle > 6d muscle | p<0.0001, F_1,149_=171.90 |
| Stable brain > stable gonad | p<0.0001, F_1,149_=150.50 |
| 6d brain > 6d muscle | p<0.0001, F_1,149_=149.05 |
| 24h brain > 24h muscle | p<0.0001, F_1,149_=138.08 |
| Stable muscle > 24h muscle | p<0.0001, F_1,149_=133.08 |
| Stable brain > 6d brain | p<0.0001, F_1,149_=108.60 |
| 6d gonad > 6d muscle | p<0.0001, F_1,149_=108.31 |
| Stable brain > stable muscle | p<0.0001, F_1,149_=104.28 |
| Alpha stable > alpha 6d | p<0.0001, F_1,149_=103.37 |
| Beta stable > beta 6d | p<0.0001, F_1,149_=92.78 |
| Stable brain > 24h brain | p<0.0001, F_1,149_=89.30 |

**Table S4:** Linear contrasts between stable males and females in sex changing groups (continued).

| 24h gonad > 24h muscle | p<0.0001, F_1,149_=88.27 |
| --- | --- |
| Beta gonad > beta muscle | p<0.0001, F_1,149_=73.48 |
| Alpha stable > alpha 24h | p<0.0001, F_1,149_=69.79 |
| Alpha brain > alpha gonad | p<0.0001, F_1,149_=66.28 |
| Beta stable > beta 24h | p<0.0001, F_1,149_=64.42 |
| Alpha gonad > alpha muscle | p<0.0001, F_1,149_=37.95 |
| Beta brain > beta gonad | p<0.0001, F_1,149_=23.28 |
| Beta gonad > alpha gonad | p=0.0009, F_1,149_=11.42 |
| Stable gonad > stable muscle | p=0.0357, F_1,149_=4.49 |
| 6d brain > 6d gonad | p=0.0364, F_1,149_=4.46 |
| ***11-Ketotestosterone*** |  |
| Alpha brain > alpha muscle | p<0.0001, F_1,152_=344.80 |
| 6d brain > 6d muscle | p<0.0001, F_1,152_=283.61 |
| Beta brain > beta muscle | p<0.0001, F_1,152_=257.79 |
| 24h brain > 24h muscle | p<0.0001, F_1,152_=249.65 |
| Alpha brain > alpha gonad | p<0.0001, F_1,152_=153.96 |
| 6d brain > 6d gonad | p<0.0001, F_1,152_=140.27 |
| Beta brain > beta gonad | p<0.0001, F_1,152_=136.33 |
| Stable brain > stable muscle | p<0.0001, F_1,152_=93.62 |
| 24h brain > 24h gonad | p<0.0001, F_1,152_=89.94 |
| Stable brain > stable gonad | p<0.0001, F_1,152_=66.78 |
| Alpha gonad > alpha muscle | p<0.0001, F_1,152_=36.98 |
| 24h gonad > 24h muscle | p<0.0001, F_1,152_=36.91 |
| 6d gonad > 6d muscle | p<0.0001, F_1,152_=26.53 |
| 6d brain > stable brain | p<0.0001, F_1,152_=20.32 |

**Table S4:** Linear contrasts between stable males and females in sex changing groups (continued).

| Beta gonad > beta muscle | p<0.0001, F_1,152_=19.53 |
| --- | --- |
| 24h brain > stable brain | p<0.0001, F_1,152_=16.07 |
| Alpha brain > beta brain | p=0.0008, F_1,152_=11.69 |
| Stable muscle > 6d muscle | p=0.0051, F_1,152_=8.08 |
| Alpha 6d > beta 6d | p=0.0072, F_1,152_=7.43 |
| Stable muscle > 24h muscle | p=0.0102, F_1,152_=6.77 |
| Alpha gonad > beta gonad | p=0.0143, F_1,152_=6.15 |
| 24h gonad > stable gonad | p=0.0287, F_1,152_=4.88 |
| Alpha stable > beta stable | p=0.0339, F_1,152_=4.58 |

List of all linear contrasts that are significant or show a trend towards significant values when comparing hormone levels of alpha and beta females at 24 hours and 6 days after male removal with those respectively of males and alpha females in stable groups. In this table the word “alpha stable” refers to males because they have alpha status, the word “beta stable” refers to alpha in stable groups because they are beta to the male.
