# Supplementary material for: Differential Responses of Brain, Gonad and Muscle Steroid Levels to Changes in Social Status and Sex in a Sequential and Bidirectional Hermaphroditic Fish
Source: PLoS One. 2012 Dec 10;7(12):e51158. doi: 10.1371/journal.pone.0051158 (PMC3519529; doi:10.1371/journal.pone.0051158)
Supplement: Table S5 — Three-way linear contrasts comparing males and females. (DOC) [file pone.0051158.s008.doc]

**Table S5:** Three-way linear contrasts comparing males and females.

|  |  |  | Stable Groups | | | | | | 24h Groups | | | | | | | 6d Groups | | | | | | |
| --- | --- | --- | --- | --- | --- | --- | --- | --- | --- | --- | --- | --- | --- | --- | --- | --- | --- | --- | --- | --- | --- | --- |
|  |  |  | α | | | β | | | α | | | β | | | | α | | | | β | | |
|  |  |  | B | G | M | B | G | M | B | G | M | B | G | M | B | | G | M | B | | G | M |
| Stable | α | B |  |  |  |  |  |  |  |  |  |  |  |  |  | |  |  |  | |  |  |
|  |  | G | 0.308  0.580 |  |  |  |  |  |  |  |  |  |  |  |  | |  |  |  | |  |  |
|  |  | M | 53.4  <0.0001 | 45.6  <0.0001 |  |  |  |  |  |  |  |  |  |  |  | |  |  |  | |  |  |
|  | β | B | 6.216  0.014 |  |  |  |  |  |  |  |  |  |  |  |  | |  |  |  | |  |  |
|  |  | G |  | 99.04  <0.0001 |  | 45.5  <0.0001 |  |  |  |  |  |  |  |  |  | |  |  |  | |  |  |
|  |  | M |  |  | 45.59  <0.0001 | 9.21  0.003 | 99.1  <0.0001 |  |  |  |  |  |  |  |  | |  |  |  | |  |  |
| 24h | α | B | 4.183  0.043 |  |  | 0.191  0.663 |  |  |  |  |  |  |  |  |  | |  |  |  | |  |  |
|  |  | G |  | 128.4  <0.0001 |  |  | 5.893  0.016 |  | 78.3  <0.0001 |  |  |  |  |  |  | |  |  |  | |  |  |
|  |  | M |  |  | 28.90  <0.0001 |  |  | 1.430  0.234 | 13.6  0.0003 | 147.5  <0.0001 |  |  |  |  |  | |  |  |  | |  |  |
|  | β | B | 6.633  0.011 |  |  | 0.006  0.936 |  |  | 0.267  0.606 |  |  |  |  |  |  | |  |  |  | |  |  |
|  |  | G |  | 117.2  <0.0001 |  |  | 0.728  0.395 |  |  | 2.770  0.098 |  | 56.5  <0.0001 |  |  |  | |  |  |  | |  |  |
|  |  | M |  |  | 38.5  <0.0001 |  |  | 0.133  0.716 |  |  | 0.657  0.419 | 11.5  0.0009 | 119.0  <0.0001 |  |  | |  |  |  | |  |  |
| 6d | α | B | 9.579  0.002 |  |  | 0.441  0.508 |  |  | 1.182  0.279 |  |  | 0.343  0.559 |  |  |  | |  |  |  | |  |  |
|  |  | G |  | 122.21  <0.0001 |  |  | 1.907  0.169 |  |  | 1.269  0.262 |  |  | 0.306  0.581 |  | 49.8  <0.0001 | |  |  |  | |  |  |
|  |  | M |  |  | 27.70  <0.0001 |  |  | 2.218  0.139 |  |  | 0.064  0.800 |  |  | 1.178  0.280 | 25.2  <0.0001 | | 155.2  <0.0001 |  |  | |  |  |
|  | β | B | 16.25  <0.0001 |  |  | 2.045  0.395 |  |  | 3.527  0.062 |  |  | 1.817  0.180 |  |  | 0.498  0.482 | |  |  |  | |  |  |
|  |  | G |  | 219.34  <0.0001 |  |  | 25.47  <0.0001 |  |  | 4.717  0.031 |  |  | 17.80  <0.0001 |  |  | | 12.693  0.0005 |  | 109.9  <0.0001 | |  |  |
|  |  | M |  |  | 49.56  <0.0001 |  |  | 0.084  0.774 |  |  | 2.180  0.142 |  |  | 0.416  0.520 |  | |  | 3.159  0.078 | 18.5  <0.0001 | | 211.4  <0.0001 |  |

Estradiol showed a significant 3-way interaction so we performed 3-way linear contrasts. Within each cell of the table, the F-value is shown on top, p-value on bottom, all F-values have df = 1, 145; yellow cells indicate that the contrast is significant. Stable alpha = male; stable beta = alpha female; in 24 hours and 6 days groups, alpha = sex-changing alpha female; beta = beta female rising to alpha status; B = brain, G = gonad, M = muscle.
