# Supplementary material for: Differential Responses of Brain, Gonad and Muscle Steroid Levels to Changes in Social Status and Sex in a Sequential and Bidirectional Hermaphroditic Fish
Source: PLoS One. 2012 Dec 10;7(12):e51158. doi: 10.1371/journal.pone.0051158 (PMC3519529; doi:10.1371/journal.pone.0051158)
Supplement: Methods S1 — (DOC) [file pone.0051158.s009.doc]

**SUPPORTING INFORMATION**

**METHODS**

***Hormone validations***

We generated a hormone pool for each of the tissue types (brain, muscle, gonad) using samples from 15 non-experimental animals. Hormone was extracted from the non-experimental tissues using the same protocol as described in the main text; the hormone residues were re-suspended in 95% EIA buffer and 5% ethanol and combined to give a pool for each tissue type. The pool was serially diluted to determine parallelism with the Cayman Chemicals, Inc. EIA kit standard curves. Briefly, 400 µl of pool was mixed with an equal volume of EIA buffer to give a 1:2 dilution; 400 µl of 1:2 was mixed with an equal volume of EIA buffer to give 1:4, and so on until 1:64. Serial dilution curves for all hormones and tissue types were parallel to the standard curve (slope comparisons: Zar 1996, p. 355): E_2_, brain: t_12_=0.016, p=0.988, gonad: t_11_=-2.163, p=0.053, muscle: t_12_=-0.065, p=0.949; T, brain: t_12_=0.232, p=0.821, gonad: t_12_=0.157, p=0.878, muscle: t_12_=0, p=1; KT, brain: t_12_=0.2, p=0.845, gonad: t_12_=0, p=1, muscle: t_12_=0.021, p=0.984. We also validated the Cayman Chemicals, Inc. EIA kits with a recovery (cold spike) assay where the tissue-extracted pool was mixed with an equal volume of each kit standard. Expected concentrations were calculated based on the known concentration of the pool. The slopes of the observed vs. expected hormone concentration regressions were all close to 1.0, indicating adequate recovery (E_2_: brain: F_1,6_=9295.6, p<0.0001, β=1.086, min recovery=110%, gonad: F_1,6_=725.1, p<0.0001, β=1.633, min recovery=90%, muscle: F_1,6_=833.1, p<0.0001, β=1.291, min recovery=112%; T: brain: F_1,6_=1023.4, p<0.0001, β=0.758, min recovery=53%, gonad: F_1,6_=107.7, p<0.0001, β=0.869, min recovery=78%, muscle: F_1,6_=469.6, p<0.0001, β=0.935, min recovery=81%; KT: brain: F_1,6_=124.4, p<0.0001, β=0.846, min recovery=93%, gonad: F_1,6_=999.9, p<0.0001, β=0.818, min recovery=81%, muscle: F_1,6_=487.1, p<0.0001, β=0.759, min recovery=93%). Seven plates were run for each hormone. Intra-assay coefficients of variation for E_2_ ranged from 3.97% to 19.7% (median=9.55%) with an inter-assay coefficient of variation of 14.8%. Intra-assay coefficients of variation for T ranged from 5.23% to 14.5% (median=5.5%) with an inter-assay coefficient of variation of 9.86%. Intra-assay coefficients of variation for KT ranged from 1.6% to 13.9% (median=8.1%) with an inter-assay coefficient of variation of 11.2%. Detection limit of the EIA kits were 8 pg/ml for E_2_, 6 pg/ml for T, 1.3 pg/ml for KT.

**RESULTS**

***Hormones in sex changing groups: Comparing with males***

All results in this section refer to Table S4 unless otherwise noted while a summary of the three-way interaction for E_2_ is reported in Table S5.

Estradiol

All main effects and their interactions were significant for E_2_ (Table 1; Table S5). As in the previous section, the three-way interaction was driven largely by differences in E_2_ among tissue types (Fig. 2). Linear contrasts revealed significantly higher E_2_ concentrations in the gonad than in brain and muscle, and in brain than muscle for all animals and at all time points (stable, 24h, 6d). Gonad and muscle E_2_ were significantly higher in animals from the sex-changing groups relative to stable groups (Fig. 2). Betas rising to the alpha position had significantly greater E_2_ (across all tissues) at 6d following male removal than in stable groups (Fig. 2). Males had significantly lower E_2_ (across all tissues) than sex-changing females and dominant females from the stable groups (Fig. 2). Overall, beta females rising to alpha position and alpha females of stable groups had significantly higher gonad and muscle E_2_ than alphas sex-changing females and males.

Androgens

There were significant status, tissue, and time × tissue effects for both T and KT (Table 1). Status differences were the result of sex-changing females and beta rising to alpha having significantly higher gonad T and higher brain KT than males and dominant female in stable group (Fig. 2). Regarding tissue differences, T and KT of all animals were significantly higher in brain than in gonad; this effect was evident mainly in stable groups for T but across all time points for KT (Fig. 2). Both androgens were higher in brain than muscle across all time points. Gonad KT was higher than muscle following male removal but not in stable groups, while gonad T was higher than muscle at all time points (Fig. 2).

Males had significantly higher T than sex changing females, but there were no significant differences in T of sex-changing alphas 24h and 6d after male removal (status × tissue; Table 1; Fig. 2). Alphas in stable groups also had significantly higher T than rising betas in sex changing groups, but there were no significant differences in T of betas 24h and 6d after male removal. Six days after male removal alpha females had higher KT than betas (Fig. 2).
